# *BRAF*^*V600E*^ patient derived colon cancer organoids identify biomarkers of response to EGFR and BRAF inhibition and replicate clinical data

**DOI:** 10.1186/s13046-026-03699-2

**Published:** 2026-03-25

**Authors:** Anna Kotarac, Hiroki Osumi, Alexander Malt, Keziban Merve Alp, Armin Jarosch, Johannes Werner, Manuela Benary, Christopher C. M. Neumann, Ryoji Yao, Philipp Mertins, Roland Eils, Dieter Beule, Christine Sers, Ulrich Keilholz, Naveed Ishaque, Sebastian Stintzing, Loredana Vecchione

**Affiliations:** 1 Charité Comprehensive Cancer Center, Charité - Universitätsmedizin Berlin, Berlin, Germany; 2 Department of Hematology, Oncology, and Cancer Immunology, (CCM), Charité - Universitätsmedizin Berlin, Berlin, Germany; 3 German Cancer Consortium (DKTK), Partner Site Berlin, Germany; 4 German Cancer Research Center (DKFZ), Heidelberg, Germany; 5 Department of Gastroenterology, Cancer Institute Hospital, Japanese Foundation for Cancer Research, Tokyo, Japan; 6 Berlin Institute of Health at Charité – Universitätsmedizin Berlin, Center of Digital Health, Berlin, Germany; 7 Max-Delbrück-Centrum Für Molekulare Medizin (MDC), Berlin, Germany; 8 Department of Pathology, Charité - Universitätsmedizin Berlin, Berlin, Germany; 9 BIH-Core Unit Bioinformatics, Charité - Universitätsmedizin Berlin, Berlin, Germany; 10 Department of Cell Biology, Cancer Institute, Japanese Foundation for Cancer Research, Tokyo, Japan; 11 National Center for Tumor Diseases (NCT), NCT Charité, Berlin, Germany; 12 Berlin Institute of Health at Charité - Universitätsmedizin Berlin, BIH Biomedical Innovation Academy, BIH Charité (Junior) (Digital) Clinician Scientist Program, Charitéplatz 1, 10117 Berlin, Germany

**Keywords:** *BRAF*^*V600E*^ mutated Colorectal cancer, Patient derived organoids, Drug resistance, Encorafenib and cetuximab, BRAF, PI3K, PTEN, AKT, ROS pathway

## Abstract

**Background:**

Drug resistance and lack of predicting biomarkers are a major challenge for cancer therapy. The combination of a BRAF inhibitor (BRAFi) together with an anti-EGFR inhibitor (EGFRi) represents a standard of care approach in *BRAF*^*V600E*^ metastatic colorectal cancer (mCRC) patients. However, predictive biomarkers of sensitivity, that could support patient selection for treatment with this combination, are currently missing. Therefore, our goal is to identify those biomarkers associated with response to the combination of BRAFi and EGFRi.

**Methods:**

Here, we established a living biobank of *BRAF*^*V600E*^ colorectal cancer patients derived organoids (PDOs) and categorized them as sensitive or resistant to the combination of BRAFi and EGFRi using short term proliferation assays. To elucidate biomarkers of response, drug testing was integrated with genomic, transcriptomic, proteomic and single-cell transcriptmic profiling of our PDOs.

**Results:**

Here we revealed the PTEN/PIK3CA/p-AKT axis as mechanism of primary sensitivity while ROS pathway inhibition as driver for primary resistance. Finally, we newly discovered histology and cellular composition as biomarker of drug response.

**Conclusion:**

These data align with recently published clinical trial data, thus reinforcing the proof that PDOs can be used for biomarker identification. The use of histology and cellular compositions as biomarkers has to be further validated in clinical setting.

**Supplementary Information:**

The online version contains supplementary material available at 10.1186/s13046-026-03699-2.

## Introduction

Colorectal cancer (CRC) is the third most common malignancy and the second deadliest cancer, with an estimated 3.2 million new cases per year projected by 2040 [1]. Over the past decade, molecular testing of metastatic CRC (mCRC) for *RAS, BRAF*, deficient mismatch repair (dMMR)/microsatellite instability (MSI) has become standard practice, guiding the selection of first-line therapies [2]. The *BRAF*^*V600E*^ mutation arises in 8–10% of CRC patients and is frequently associated with distinct clinical features, including female, hypermethylation, MSI-High (MSI-H), limited chromosomal instability (CIN), worse survival after relapse (SAR), and poor overall survival [3–5]. According to the National Cancer Network (NCCN) and European Society of Medical Oncology (ESMO) guidelines, the administration of 5-Fluorouracil (5-FU) or fluoropyrimidines in dual and triple combination with either oxaliplatin and/or irinotecan (FOLFOX, FOLFIRI, FOLFOXIRI) is the mainstay of first-line treatment of patients with *BRAF*^*V600E*^ microsatellite stable (MSS) mCRC [6, 7]. Notably, approximately 30% of *BRAF*^*V600E*^ cases are MSI-H, for which immunotherapy has emerged as the standard of care in first-line setting [8].

While BRAF and MEK inhibitors (BRAFi and MEKi) have revolutionized the outcome in other solid and non-solid *BRAF*^*V600E*^ mutated tumors, their efficacy in *BRAF*^*V600E*^ mCRC has been limited [9, 10]. The unresponsiveness of *BRAF*^*V600E*^ mCRC to BRAFi was uncovered by Prahallad et al. through a synthetic lethal screen, which identified Epidermal growth factor receptor (EGFR) inhibition as a lethality in combination with BRAFi [11]. This discovery led to the design of several clinical trials in which *BRAF*^*V600E*^ mCRC patients were treated with dual combinations of BRAFi and EGFR inhibitors (EGFRi) and the addition of MEKi or PI3K inhibitors (PI3Ki) [12, 13]. The largest clinical trial of *BRAF*^*V600E*^ mCRC to date, the BEACON phase III trial, demonstrated that EGFRi + BRAFi and EGFRi + BRAFi + MEKi significantly improved overall survival (OS) and progression-free survival (PFS) compared to the standard of care in second- and third-line settings. However, a proportion of patients either do not respond or derive limited clinical benefit, thus highlighting the need to discover both mechanisms of primary and secondary resistance to these combinations [14].

Patient-derived organoids (PDOs) are three-dimensional in vitro cultures of normal and/or tumor tissues with multiple cell lineages, that recapitulate cellular heterogeneity, maintain the self-organizing capacities of cells, and preserve the histopathological and molecular features of parental tissue [15]. Isolation of PDOs from human primary tumors and metastases has enabled the establishment of living biobanks [16, 17]. PDOs have been shown to represent the molecular features and morphology of the tissue of origin, thus enabling functional experiments such as ex-vivo drug testing [17–20]. While PDOs have been widely adopted for preclinical research, their potential role to predict and reproduce treatment responses and discover biomarkers in real-world or clinical trial data remains unexplored.

In this study we demonstrate that *BRAF*^*V600E*^ CRC PDOs represent a valid tool to replicate clinical trial data [21]. In particular, by using a cohort of PDOs generated from patients with *BRAF*^*V600E*^ mutated CRC, we identified similar features of mechanisms of resistance to the combination of cetuximab and encorafenib, as well as features of intrinsic sensitivity. Moreover, we identified new potential biomarkers of cetuximab and encorafenib sensitivity that may guide future patient stratification for targeted therapies. Finally, our findings suggest that histology and cellular composition might play a role in primary resistance, offering new insights into the heterogeneity of the treatment response.

## Methods

Methods are summarized in Additional File 1.

## Results

### Clinical features and responsiveness to erlotinib and encorafenib of BRAF^V600E^ colorectal cancer patient-derived organoids

To identify mechanisms of primary resistance to cetuximab and encorafenib, we established a living biobanking of ten *BRAF*^*V600E*^ CRC PDOs representing different clinical stages. The main clinical characteristics of the patients from which the PDOs models were derived are summarized in Table 1. The majority of PDOs (50%) originated from CRC with tubular histology, whereas 30% and 20% were derived from poorly differentiated and mucinous histology, respectively. Nearly all (90%) patients were female. Primary tumors were predominantly located in the transverse colon (40%), ascending colon (30%) and in the cecum (30%) with only one case in the rectum (10%). Despite the fact that 50% of patients had a stage IV metastatic disease at the time when PDOs were established, all but two PDOs were derived from untreated primary tumors.

**Table 1:** Patient characteristics

*BRAF*^*V600E*^ mutated PDO	Gender	Confirmation of *BRAF*^*V600E*^ mutation	Confirmation of *BRAF*^*V600E*^ mutation*^3^	MMR-status*^4^	Histology	Grading	Tumor location	Origin of the cohort	Organ of origin of the PDO	Tumor stage at the time of establishment	Sites of metastasis
HCT153	F	V600E*^1^	V600E	dMMR	tubular	NA	right	Japan	colon	IV	multiple LN
							(transverse)				
HCT156	F	V600E*^1^	V600E	dMMR	tubular	NA	right	Japan	colon	IIIb	NA
							(transverse)				
HCT161	F	V600E*^1^	V600E	dMMR	poorly differentiated	G3	right	Japan	colon	II	NA
							(transverse)				
HCT162	F	V600E*^1^	V600E	dMMR	tubular	NA	right	Japan	colon	IV	liver
							(cecum)				
HCT166	F	V600E*^1^	V600E	dMMR	tubular	NA	right	Japan	colon	IIIa	NA
							(ascending)				
HCT178	F	NA	V600E	dMMR	poorly differentiated	G3	right	Japan	colon	III	NA
							(cecum)				
OT212	F	V600E*^2^	V600E	pMMR	poorly differentiated	G3	right	Berlin	colon	IV	NA
							(ascending)				
OT261	F	V600E*^2^	V600E	dMMR	tubular	G1	right	Berlin	colon	I	NA
							(ascending)				
M2	F	V600E*^2^	V600E	pMMR	mucinous	G3	right	Berlin	liver	IV	liver
							(cecum)				
ICP_CRC_01	M	V600E*^2^	V600E	pMMR	mucinous	NA	right	Berlin	bronchial LN	IV	lung, LN, liver, peritoneum
							(transverse)				

*HCT* human cancer tissue, *ICP* informative Charite patient, *IHC* immunohistochemistry, *LN* lymph node, *M* Male, *dMMR* deficient DNA mismatch repair, *pMMR* proficient Mismatch Repair, *NA* not available, *OT* Oncotrack, *PDO* patient-derived organoid

^*^1 at time of PDO establishment by Sanger Sequencing, *2 at time of PDO establishment by WES, *3 at time of PDO expansion by WES and RNA Seq,

^*^4 at time of PDO expansion by IHC, dMMR: deficient DNA mismatch repair, pMMR:Proficient Mismatch Repair; CRC: colorectal cancer; F:Female;

**Fig. 1:**
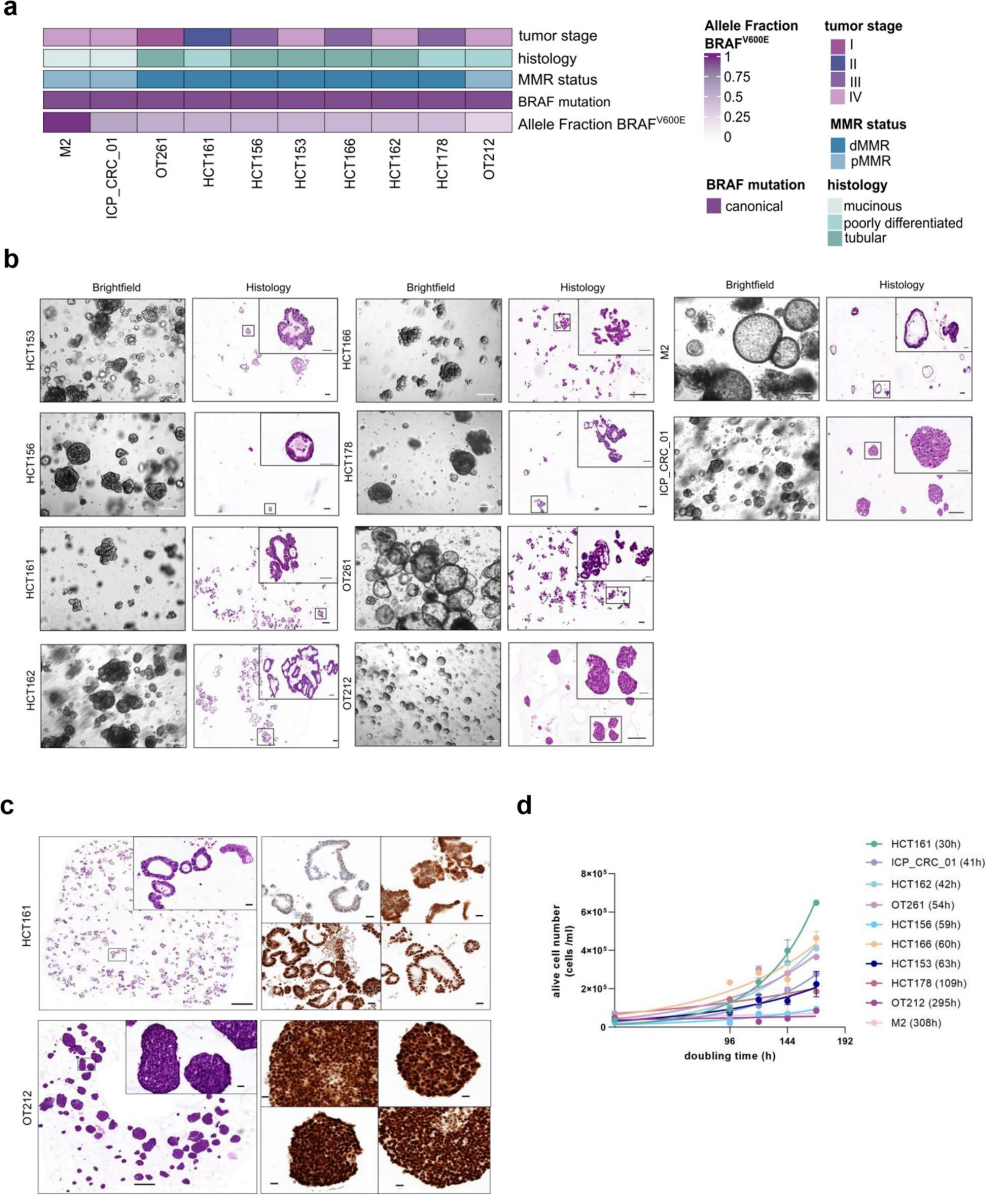
Clinical, morphological and histological features of *BRAF*^*V600E*^ CRC PDOs. **a** Oncoplot showing *BRAF*^*V600E*^ allele fractions in ten *BRAF*^*V600E*^ CRC PDOs determined by WES. Moreover, tumor stage, histology, MMR status and *BRAF* mutations are annotated. **b**, Brightfield images and Hematoxylin and Eosin (HE) staining of ten established *BRAF*^*V600E*^ CRC PDOs. Scale bar, 200 μm for the brightfield images, Scale bar histopathological images, 200 μm (big images) and 50 μm (zoomed- in images). **c**, HE-stained PDO model HCT161 with a detailed inset highlighting cellular architecture (left). Scale bar, 500 µm. Immunohistochemical analysis showing nuclear loss of MLH1 and PMS2 (right panel-top) and retained nuclear expression of MSH2 and MSH6 (right panel-bottom). Scale bar, 20 µm. HE-stained PDO model OT212 with a detailed inset highlighting cellular architecture (left panel). Scale bar, 500 µm. Immunohistochemical analysis showing retained nuclear expression of MLH1 and PMS2 (right panel-top) and MSH2 and MSH6 (right panel-bottom). Scale bar, 20 µm. **d**, Doubling time of ten established *BRAF*^*V600E*^ CRC PDOs. Alive cell number (cells/ml) was determined after 96, 120, 144 and 168 h. Each data point corresponds to three technical replicates and is presented as mean ± SEM

All PDO models were confirmed to have the *BRAF*^*V600E*^ mutation (Fig. 1a). Established models exhibited distinct morphological features. Some appeared cohesive and cystic with well-organized cellular polarity, whereas others appeared small, increasingly discohesive, or in grape-like structures, highlighting their phenotypic heterogeneity (Fig. 1b). Hematoxylin and eosin staining confirmed the cancer origin for all models. Seven out of ten models were mismatch repair deficient (dMMR) as defined by immunohistochemistry (IHC) analysis (Fig. 1c, Suppl. Table 1). Growth kinetics varied across models, with doubling times reflecting heterogeneous proliferation rates (Fig. 1d).

**Fig. 2:**
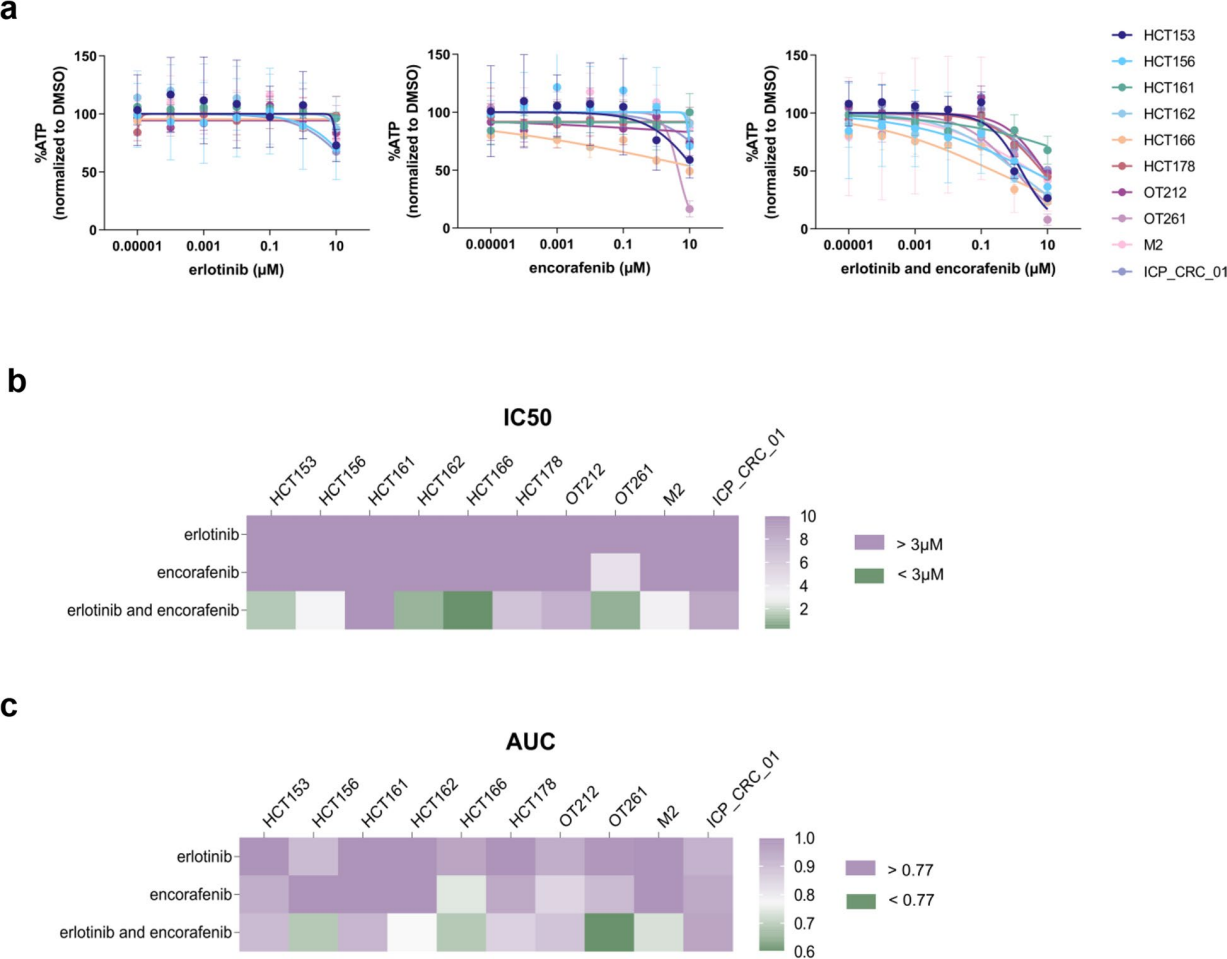
Responsiveness of *BRAF*^*V600E*^ CRC PDOs to erlotinib and encorafenib.**a**, Dose response curves of ten *BRAF*^*V600E*^ CRC PDOs treated with single agents erlotinib, encorafenib, and the combination of erlotinib and encorafenib. For each drug concentration ATP levels were assessed, normalized to DMSO. Data were normalized, transformed in logarithms and fit into dose response curve. The graphs have a linear x-axis and are labeled with antilog. Each data point is the mean of at least three independent biological replicates and error bars represent the mean ± SEM. Heatmaps report the IC50 (**b**) and AUC (**c**) values of all PDOs treated with single agents and dual combination of erlotinib and encorafenib. Purple and green color is representing IC50 values that are bigger or lower than the individual Cmax of erlotinib (3.1 µM), and encorafenib (2.9 µM), respectively. Those Cmax were used as cut-off to define sensitivity/resistance based on IC50 data. AUC cut-off values determined by the Jenks Natural Breaks classification method are > 0.77 (purple) and < 0.77 (green). Sensitive models are represented in green, intermediate in white and resistant in purple

To evaluate drug responsiveness, we treated PDOs with erlotinib (EGFRi) and encorafenib (BRAFi). Cetuximab (EGFRi) was ineffective in our cultures (data not shown), which prompted the use of erlotinib. All models were intrinsically resistant to single agents erlotinib and encorafenib (Fig. 2a); however, 40% were classified as sensitive to the combination of erlotinib and encorafenib (E + E), 20% as intermediate, and 40% as resistant, based on IC50 values (Fig. 2b). The median Z-factor score, a measure assay plate quality, across all drug testing plates was 0,71 (n = 87; upper and lower quartile = 0,84 and 0,55 respectively), consistent with an experimentally robust assay. The area under the curve (AUC) for the E + E combination further supported these findings, showing a significant positive correlation (rho = 0.79, *P* = 0.003) between the two parameters used for sensitivity classification (Fig. 2c, Suppl. Figure 1a). Additionally, neither IC50 nor AUC values correlated with doubling time (*P* > 0.05), indicating that intrinsic sensitivity or resistance was independent of the growth rate (Suppl. Figure 1b and c). Interestingly, concordant patient outcomes under cetuximab and encorafenib and organoid responses to erlotinib and encorafenib were observed in only one model. Specifically, the ICP_CRC_01 model was derived from a male patient who progressed under first-line treatment with FOLFOX plus bevacizumab and subsequently underwent a new biopsy of a bronchial lymph node for re-molecular analysis prior to initiating second-line therapy with encorafenib and cetuximab. An additional biopsy of the bronchial lymph node was obtained for organoid establishment. With a progression-free survival of 3.1 months (98 days) and progressive disease as the best observed response, the patient was classified as primarily resistant to the combination therapy. Consistently, the corresponding organoid culture also exhibited intrinsic resistance. Although limited to a single case, this example highlights the clinical reproducibility of our models. Finally, we observed a synergistic effect of the E + E dual combination in 30% of our models, whereas 60% showed an additive effect and 10% displayed antagonism (Suppl. Figure 2a-j, Suppl. table 2). These observations were independent of the sensitivity classifications based on IC50 and AUC, suggesting additional layers of complexity in the drug response mechanisms.

### Genomic profiling identifies PTEN alterations as potential biomarker of sensitivity to erlotinib and encorafenib

**Fig. 3:**
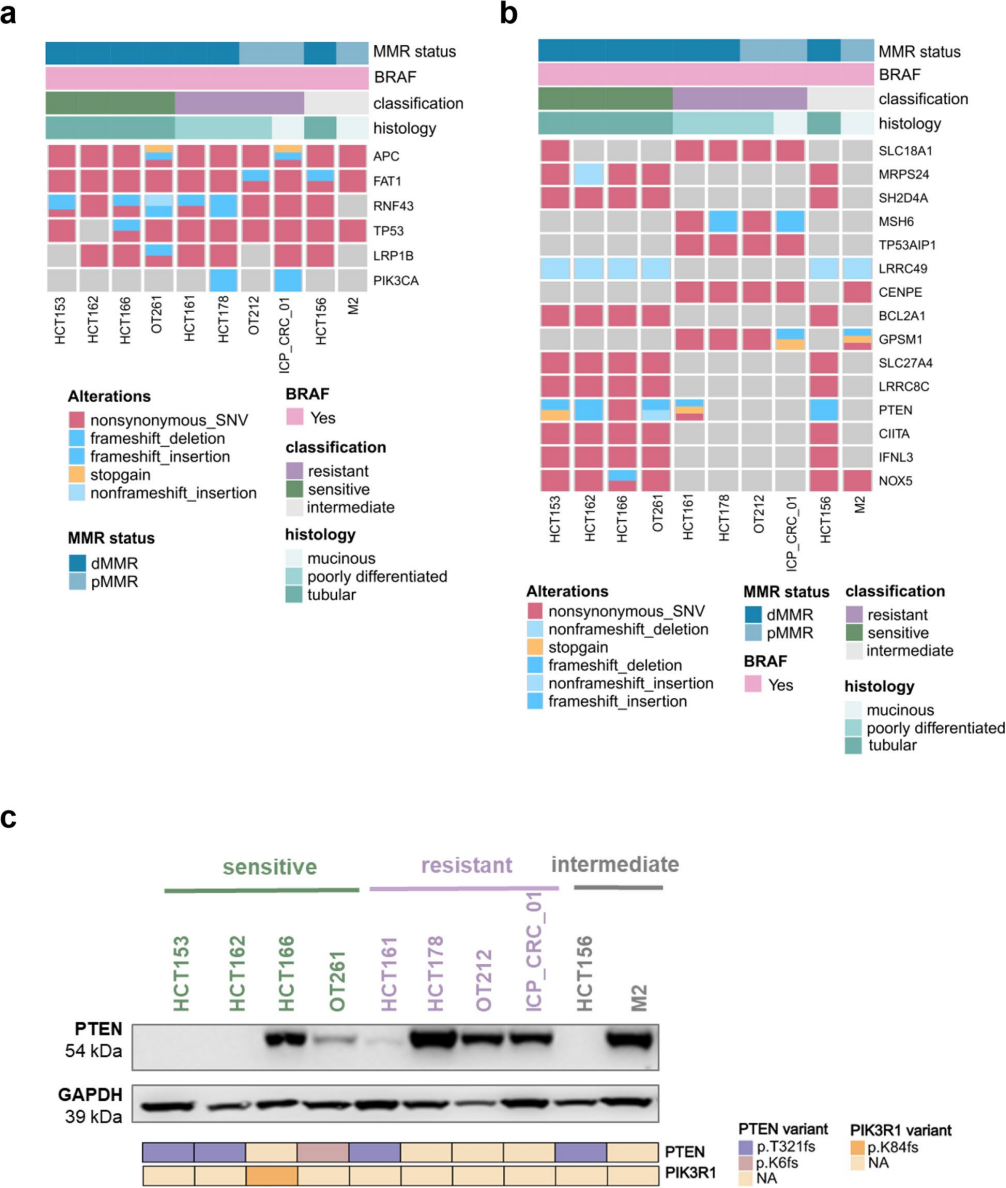
Genomic profiling of *BRAF*^*V600E*^ CRC PDOs. **a** Oncoplot showing alterations of frequent colorectal cancer co-mutations in ten *BRAF*^*V600E*^ PDO model including *APC, FAT1, RNF43, TP53, LRP1B, PIK3CA* determined by WES. MMR status, histology and classification of response of each model are annotated above. **b** Oncoplot shows alterations of genes exclusively mutated PDO models resistant or sensitive to erlotinib and encorafenib combination determined by WES. MMR status, histology and classification of response of each model are annotated above. Mutational exclusivity was analyzed by binarizing mutations (mutated = 1; not mutated = 0) and performing Fisher’s exact T-test to compare resistant versus sensitive samples. **c** Western Blot of baseline PTEN expression in ten *BRAF*^*V600E*^ CRC PDOs. Models are annotated as sensitive (green), resistant (purple) and intermediate (grey). GAPDH was used as loading control. Matching Oncoplot showing alterations of *PTEN* and *PIK3R1* in each model is displayed below

To identify genomic alterations predictive of responsiveness to the E + E combination, PDOs underwent whole exome sequencing (WES) at baseline. No significant association was identified between treatment response and mutations in cancer-associated genes commonly co-mutated in CRC (Fig. 3a). In particular, *APC* and *FAT1* were found to be mutated in all models, while *RNF43* and *TP53* were mutated in 90% of PDOs and *LRP1B* in 70%. Next, we identified genes that were significantly exclusively mutated in either the sensitive or resistant models (Fisher exact test with BH correction, *P* < 0.05). *PTEN, NOX5, BCL2A1, SLC27A4, LRRC8C, CIITA*, and *IFNL3* were exclusively mutated in sensitive models, whereas *SCL18A1, MSH6, TP53AIP1, CENPE*, and *GPSM1* were exclusively mutated in resistant models (Fig. 3b). To explore the functional implications of these mutations, we examined their expression at the RNA level. However, no statistically significant association between mutations and expression at the RNA level was observed for any of the aforementioned genes (Suppl. Table 3). Notably, two out of the four resistant models harboured pathogenic *MSH6* mutations. Nevertheless, no association with protein loss and therefore MMR status was found (Suppl. Table 1). In contrast, *PTEN* mutations were associated with protein loss (Fig. 3c), suggesting that protein-level assessment may be more informative, in some cases, than RNA expression for predicting phenotypic outcomes of genomic mutations. More importantly, all models sensitive to E + E show mutually exclusive *PTEN* alterations (mutations or loss) or mutation in *PIK3R1*, a negative regulator of the PIK3CA pathway, thus opening the possibility that PTEN loss as well as mutations in *PIK3CA* negative regulators such as *PIK3R1* might be used as a biomarker of sensitivity to E + E combination therapy.

Finally, we examined the publicly available TCGA database to comprehensively analyze the differential mutations identified in resistant or sensitive models and their association with *BRAF*^*V600E*^ mutation in real-world data. We found *NOX5, PTEN, MSH6, CIITA, CENPE* and *GPSM1* to be significantly co-occurrent with the *BRAF*^*V600E*^ mutation in CRC. In particular among the mutated genes in the sensitive group, we found *NOX5* to occur in 7%, *PTEN* in 15%, *CIITA* in 14% of *BRAF*^*V600E*^ CRC patients. Regarding the genes mutated in the resistant group, *MSH6*, *CENPE* and *GPSM1* were mutated in 11%, 14% and 5% of *BRAF*^*V600E*^ CRC patients, respectively (Suppl. Table 4).

### Proteomics and Phosphoproteomics analysis identifies p-AKT, EGFR signaling and cell cycle pathways as biomarkers of sensitivity to erlotinib and encorafenib

**Fig. 4:**
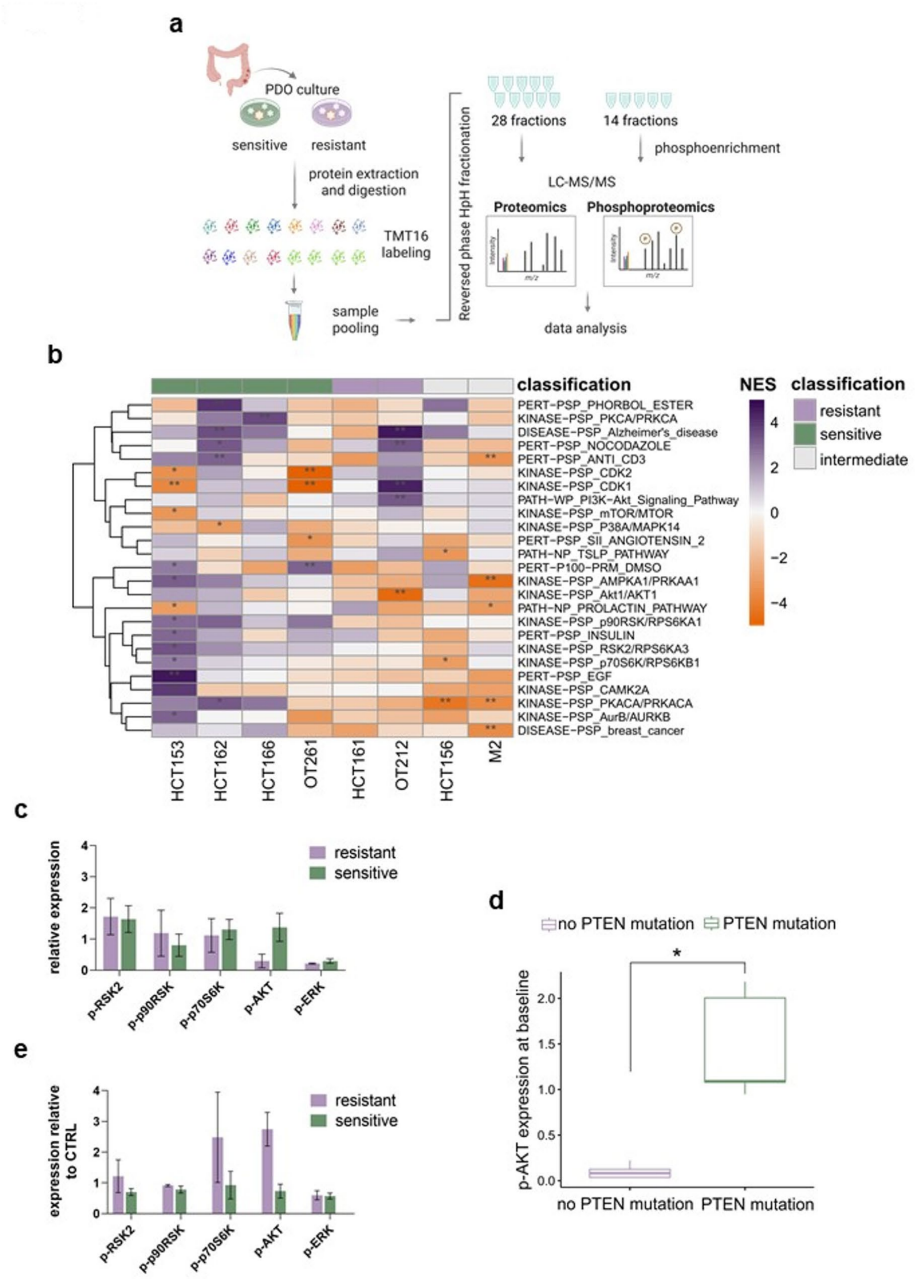
Differential proteomic signatures associated with E + E response. **a** Schematic overview of mass spectrometry workflow. **b** Heatmap displays normalized enrichment scores (NES) of significantly enriched pathways in the single sample GSEA (ssGSEA) analysis of eight *BRAF*^*V600E*^ CRC PDOs at baseline using PTMsigDB database. Normalized reporter ion intensities are used for ssGSEA analysis. Pathways that are significantly enriched at least in one sample are selected. False Discovery Rate (FDR) levels are shown with asterisks. Columns are annotated and ordered by drug response classification. **c** Relative expression of p-RSK2, p-P90, p-P70, p-AKT and p-ERK expression at baseline between four sensitive (green) and four resistant models (purple) determined by Western Blot (mean from four different biological replicates/samples ± SEM). **d** Bar graph shows p-AKT expression of ten *BRAF*^*V600E*^ PDOs in samples without *PTEN* mutation (purple) and with *PTEN* mutation (green). * P < *0.05,* Wilcoxon-Mann–Whitney test. **e**, Relative quantified expression of p-RSK2, p-P90, p-P70, p-AKT and p-ERK in sensitive and resistant models treated with E + E (3.1 µM erlotinib + 2.9 µM encorafenib) relative to control (DMSO) determined by Western Blot. Green bars show the mean ± SEM of three sensitive models and purple bars the mean ± SEM of two resistant models

To uncover functional correlates between baseline proteomic expression and treatment response, liquid chromatography-tandem mass spectrometry (LC–MS/MS) was performed (Fig. 4a). Among the analyzed models, four were categorized as sensitive, two as resistant, and two as intermediate. Single sample Gene Set Enrichment Analysis (ssGSEA) using the PTMsigDB database did not identify statistically significantly enriched pathways when examining the total proteomic data (data not shown). In contrast, phosphoproteomics analysis identified significant enrichment of kinase activity in sensitive models, including kinases PKCA, CDK1, AMPKA1, AKT1, p90RSK, RSK2, p70S6K, EGF, and PKACA (Fig. 4b). To better understand the interaction among these kinases, we further performed pathway analysis using Enrichr [22–24]. Among the ten most significant Reactome pathways, we found the CREB phosphorylation pathway, RSK activation, ERK MAPK targets, and Signaling by Receptor Tyrosine Kinases (RTK) (Suppl. Table 5). Furthermore, among the ten most significant pathways from BioPlanet 2019, we found EGFR pathway, EGF/EGFR signaling pathway, PI3K pathway, Kit receptor signaling pathway, mTOR signaling and TGF-beta signaling pathway (Suppl. Table 6). These findings indicate that elevated levels of activated EGFR/MAPK signaling and the CREB pathway may serve as predictive biomarkers of the response to E + E. In particular, CREB is an important transcriptional factor that regulates the expression of several proteins, including c-Jun and Cyclin D1 [25]. The latter is important for progression through the G1 phase of the cell cycle, and its protein expression is rapidly degraded as cells enter the S phase. Inhibition of EGFR by cetuximab promotes arrest in the G1 phase of the cell cycle and stimulates apoptosis by blocking EGFR-activated survival pathways [26]. This would, therefore, support our findings that higher expression of cell cycle proteins may correspond to responsiveness through EGFR inhibition.

To validate these observations, Western Blot analysis of the identified proteins/pathways was performed on PDOs at baseline. While no significant differences were observed in p-RSK2, p-p90RSK, and p-p70S6K expression, the mean baseline expression of p-AKT was approximately seven times higher in sensitive models as compared to resistant models (Wilcoxon-Mann–Whitney test, *P* = 0.05) (Fig. 4c, Suppl. Figure 3a). Given the enrichment of *PTEN* alterations in sensitive models, we investigated whether p-AKT upregulation was associated with PTEN loss. Indeed, p-AKT expression was significantly upregulated in models with *PTEN* mutation/loss (Wilcoxon-Mann–Whitney Test, *P* = 0.0079) (Fig. 4d), suggesting that both *PTEN* alterations and baseline p-AKT levels may be considered as predictive biomarkers of sensitivity to E + E. To explore dynamic changes in the aforementioned proteins upon treatment, we measured protein expression in three sensitive, two resistant and one intermediate model after 24 h of E + E exposure. Interestingly, a different phenotype was observed. In particular, the mean relative expression of p-AKT in the two resistant models was approximately three times higher than in sensitive models (Wilcoxon-Mann–Whitney test, *P* = 0.2) (Fig. 4e, Suppl. Figure 3b-c). To test whether the observed increase in p-AKT in resistant models was functionally inducing resistance, two resistant and one intermediate model were treated with either the single agent PI3K inhibitor alpelisib or the single agent pan-AKT inhibitor (pan-AKTi) capivasertib or the combination with E + E with either fixed concentration of alpelisib (E + E + A) or capivasertib (E + E + C) (Suppl. Figure 3d-h). The results show that both triple combinations are more potent than E + E in resistant models. In particular, while for resistant model OT212 and intermediate model HCT156 the resistant phenotype can be reversed with both E + E + A and E + E + C, the resistant model HCT161 shows better benefit from the combination of E + E + C. This could probably be due to its high pAKT expression level at baseline which increases even further upon treatment with E + E. (Suppl. Figure 3 A, C). In summary, this indicates that the addition of a either a PIK3Ki or apan-AKTi to E + E in resistant models may reverse their resistant phenotype.

### Integration of protein and gene expression analysis identifies ROS pathway as potential predictor of resistance to erlotinib and encorafenib

**Fig. 5:**
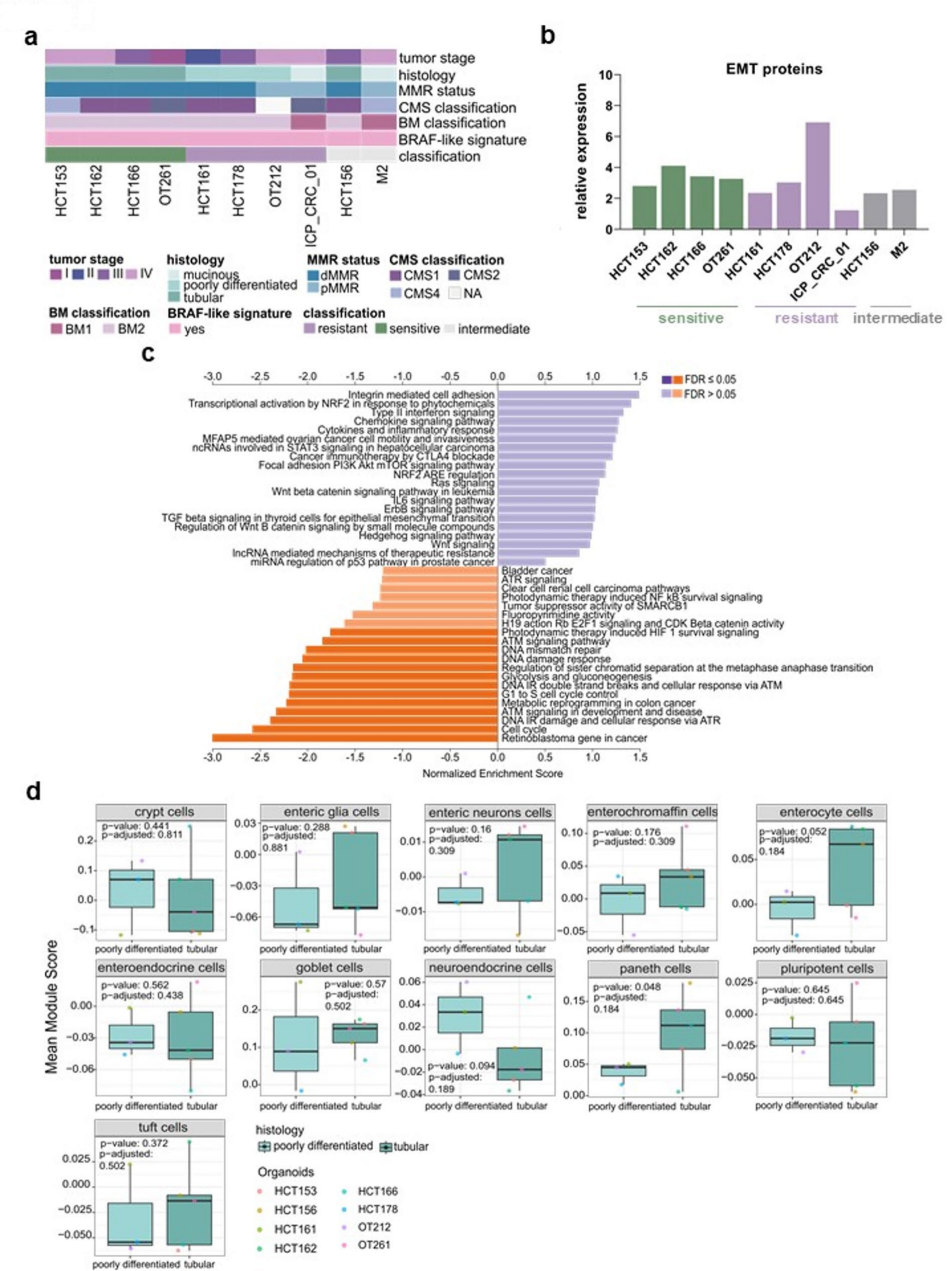
Multi-omic integration reveals predictors for E + E response. **a** Annotation of tumor stage, histology, CMS classification, BM classification, *BRAF*-like signature and classification of resistant, sensitive and intermediate models for each *BRAF*^*V600E*^ CRC PDO. **b** Mean relative expression of EMT proteins including total and phospho SMAD2, vimentin, SMAD4 and N-cadherin at baseline in ten *BRAF*^*V600E*^ CRC PDO models. Green bars represent the expressions in sensitive models, purple bars represent the expression in resistant models and grey bars the expression in intermediates models. **c **Bar plot for enriched pathways from post- treatment GSEA analysis of three sensitive *BRAF*^*V600E*^ PDOs. FDR < 0.05 (dark orange) and > 0.05 (light orange and purple). **d** Boxplots showing the mean module score of intestinal cell types in poorly differentiated models (resistant) vs. tubular (sensitive) models. Gene lists for module score calculations were obtained from PanglaoDB [36], representing cell-type-specific gene sets. Module scores were calculated using the Seurat function AddModuleScore. Reported are both *p*-values and *p*-adjusted

To evaluate whether gene expression profiles were associated with response to E + E, we performed bulk RNAseq on all models at baseline, as well as on three sensitive, two resistant and one intermediate model following treatment. Unsupervised hierarchical clustering of the 2,000 most variably expressed genes did not reveal distinct clusters associated with sensitivity or resistance, either at baseline or post-treatment (Suppl. Figure 4a). To further investigate whether linear combinations of gene expressions could associate with response, we applied Principle component analysis (PCA), which revealed that principle component 6 (PC6, explaining 5.6% of variation) significantly correlated with E + E IC50 (Spearman correlation, FDR = 0.021). Furthermore using multivariate ANOVA, we found that PC6 also associated with the histology (ANOVA, Eta squared = 0.076, FDR = 0.020) (Suppl. Figure 4b). Consistent with this, a significant association was observed between histology and response. Indeed, all models with tubular histology were sensitive, while all poorly differentiated and/or mucinous were resistant or intermediate (Fig. 5a).

We further classified PDOs into Consensus Molecular Subtypes (CMS) [27], BRAF-like [28], and BRAF mutant (BM) [29] subtypes based on transcriptomic profiles (Fig. 5a). All models were classified as BRAF-like, indicating that this signature could not help predicting the response to E + E. Among the CMS subtypes, five models were classified as CMS1, two as CMS2, two as CMS4 and one as unclassified. Two out of ten models were classified as BM1 and of those only one was classified as CMS4. Notably, the response to E + E was independent of CMS and BM classification.

Previous reports by Middleton et al. [30] suggested that *BRAF*^*V600E*^ CRC classified as BM1 showed better PFS and OS with the combination of dabrafenib, trametinib and panitumumab compared to BM2 tumors. As reported by Barras et al. [29], the BM1 classification is mainly characterized by high levels of p-AKT, KRAS/AKT and epithelial-mesenchymal transition (EMT) markers. While we observed a clear association between p-AKT expression and sensitivity to E + E, no correlation was found with the BM classification, as mentioned previously. To better dissect the CMS and BM phenotypes and focus on EMT and/or epithelial components as surrogate markers of response, we performed gene set variation analysis (GSVA) on RNAseq data as well as Western Blot analysis of EMT markers (Fig. 5b, Suppl. Figure 4c-d). Because of their influence on EMT expression through PI3K/AKT and MAPK activation [31], PD-L1 associated genes [32] were also considered in our analysis and found to be enriched in 75% of our sensitive and 25% of our resistant models (Suppl. Figure 4c). The expression of EMT genes was equally distributed among sensitive and resistant models (Suppl. Figure 4c), while the sum of the expression of the tested EMT markers at protein level was slightly higher in sensitive models with the exception of one resistant model (Fig. 5b). The observed EMT signal at protein level was driven mainly by SMAD4 expression (Suppl. Figure 5a). Although the observed differences are not statistically significant because of the low samples size of our cohort, this might warrant further in vivo validation. Differential gene expression analysis of resistant versus sensitive models identified 26 upregulated genes in resistant models and 16 upregulated genes in sensitive models (Log2 FC > 0.5 or < −0.5 and BH adjusted *P* < = 0.01) (Suppl. Table 3) with no significant up- or downregulated pathways at basal expression between the two groups (Suppl. Figure 5b). In contrast, post-treatment pathway analysis showed significant downregulation of the retinoblastoma gene in cancer (FDR < 0.05, cell cycle (FDR < 0.05), DNA damage (FDR < 0.05), glycolysis and gluconeogenesis (FDR = 0.0008) pathways in sensitive models (Fig. 5c) but no significant differential pathways upon treatement in resistant models. These findings might indicate successful inhibition of cell proliferation upon MAPK pathway inhibition, in sensitive but not resistant models. However, glycolysis, mTORC, PIK3CA and ROS pathways were enriched upon treatment in resistant versus sensitive models based on single sample ssGSEA (Suppl. Figure 5c). The enrichment of the mTORC and PIK3CA pathways was consistent with the increase in p-AKT upon treatment in resistant versus sensitive models observed at the protein level. Moreover, among the most significantly downregulated genes upon treatment in resistant models, we identified NOX5 (logFC −3.8, BH corrected negative binomial test, *P* = 0.0006). NOX5 encodes for NAPDH oxidase and is responsible for the production of ROS (Suppl.Table 7).

To further understand the role of NOX5 and its association with the ROS pathway, which was enriched in resistant models upon treatment, we examined the expression of stress and apoptotic proteins upon treatment using phospho-arrays. We observed an increase in HIF1a and its downstream target, Carbonic Anhydrase (Suppl. Figure 5 d, Suppl. Tables 8–10), which are known negative regulators of ROS, resulting in low levels of lipid peroxidation and inhibition of ferroptosis [33]. HIF1a is known to regulate hypoxia, increase the expression of glycolytic enzymes to maintain bioenergetic homeostasis during hypoxia, and promote tumor survival and growth [34]. These findings align with our data, as resistant models exhibited increased glycolysis at the gene expression level in ssGSEA post-treatment (Suppl. Figure 5c). Additionally, array analysis revealed an increase in SIRT2 and its downstream target SOD2, also known to be negative regulators of ROS [35], and an increase in HO-2/HMOX2, which are known to be anti-apoptotic proteins in resistant models. Based on the upregulation of negative regulators of ROS at protein levels (HIF1a, Carbonic Anhydrase, SIRT2, SOD2) and downregulation of positive regulators (NOX5) at RNA level, we hypothesize that resistant models may exhibit downregulation of the ROS pathway upon treatment. This hypothesis is further supported by the reduction of phopsho-JNK, a downstream effector of ROS, at the protein level (Suppl. Figure 5 d). A schematic representation of the ROS pathway and related proteins in resistant models upon E + E treatment is presented in Suppl. Figure 6a. Concomitant with a reduction in ROS pathway, we observed an increase in anti-apoptotic proteins, such as HO-2/HMOX2 and BCL2, which may explain the survival of resistant models upon treatment. We hypothesized that inhibiting the anti-apoptotic protein BCL, we could induce cell death in resistant models. Indeed, the addition of the BCL2 inhibitor (BCLi) obatoclax to E + E slightly reduced IC50 values in two resistant models and one intermediate model (Suppl. Figure 6b-e).

In summary, while the BM, CMS and BRAF-like classification were not associated with treatment response, one of the BM1 downstream markers, such as p-AKT may predict sensitivity to E + E. With regard to EMT markers in vivo validation may be required to show significance. Moreover, the upregulation of negative regulators of ROS, as well as the downregulation of its positive regulators, upon treatment appear to happen in resistant models, and to be associated with an increased expression of anti-apoptotic proteins. The addition of a BCLi to E + E rescued the resistant phenotype by improving to a limited extent the treatment efficacy.

### Single-cell analyses reveal insights into cell-of-origin and therapy response to erlotinib and encorafenib in BRAFV600E mutated CRC PDOs

As previously described, we observed a strong association between histology and response to E + E (Fig. 5a). To explore whether histology could serve as biomarker of responsiveness, we analysed scRNA-seq data at basal expression to identify differences in cell of origin and cell type composition between sensitive and resistant models. Using Enrichr [22–24] for cell type enrichment analysis, we found that genes upregulated in sensitive models significantly overlapped with epithelial cells in intestine (Descartes Cell type, *P* < 0.01), LGR5 + stem cells (Cell Marker 2024, adjusted *P* < 0.01), Enterocytes large intestine (Cell Marker 2024, adjusted *P* < 0.01), stem cell colon (Cell Marker 2024, adjusted *P* < 0.01), paneth cells (HuMAP ASCT plus B, adjusted *P* < 0.01) and goblet cells (HuMAP ASCT plus B, adjusted *P* < 0.01). In contrast, genes upregulated in resistant models significantly overlapped with neuroendocrine cell trachea mouse (Cell Marker 2024, adjusted* P* < 0.01), enteroendocrine precursor cells intestinal crypt (Cell Marker 2024, adjusted *P* < 0.01) and neuroendocrine cell in stomach and lung (Descartes Cell, adjusted *P* < 0.01).

To further validate these findings, we calculated the mean module score of intestinal cell types [36]. Resistant models showed a trend towards enrichment with neuroendocrine cells (*P* = 0.075), a significant depletion with enteric neuron cells (*P* = 0.04) and a trend towards lower levels of enterocytes and paneth cells (*P* = 0.078 and 0.4, respectively) as compared to sensitive models (Suppl. Figure 7a). We next investigated whether these differences in cell type composition could distinguish tubular (sensitive) from poorly differentiated (resistant) models. Poorly differentiated models had less enterocytes and paneth cells (*P* = 0.052 and 0.048, respectively) but more neuroendocrine cells (*P* = 0.09) (Fig. 5d).

In summary, our data show that well-differentiated *BRAF*^*V600E*^ CRC PDOs were predominantly enriched in expression programs related to paneth cells and enterocytes, while poorly differentiated *BRAF*^*V600E*^ CRC PDOs were mainly enriched in expression programs related to neuroendocrine cells and this seems to correlate with response to E + E. Due to the limited number of our samples the observed results are upon multiple test correction formally non statistically significant. Nevertheless, when we retrospectively looked at the histological report of the tumor from which our models were derived, we identified the OT212 as a model for which histology analysis matches with scRNA subtyping. Indeed the pathological report reports a poorly differentiated adenocarcinoma of the ascending colon with partial neuroendocrine differentiation due to partial expression of synaptophysin and sparse expression of chmromogranin. Indeed, as reported in Fig. 5d and supplementary Fig. 7a, the OT212 model is the model with the highest expression of the mean module score for neuroendocrine cells. Even if based on a sigle case, this observation might indicate reproducibility of our findings and strongly suggests further investigation of histology and cell-of-origin expression signatures as biomarkers for predicting treatment outcomes in *BRAF*^*V600E*^ mutated CRC upon cetuximab and encorafenib.

## Discussion

Over the last decade, PDOs have emerged as promising models both in basic and translational research mainly due to a higher representation of tissue of origin and differentiation states in comparison to conventional cell lines. Previous studies have successfully shown the feasibility of using PDOs for small-and medium-scale drug testing and the identification of predictive molecular features of drug response [16, 17, 37]. Nevertheless, tumor organoids are still far away from being used as predictive tool for decision making since several studies failed to show a consistent value in predicting treatment response in vivo [38, 39].

In order to identify clinically applicable biomarkers of response to cetuximab and encorafenib in *BRAF*^*V600E*^ CRC, we established a living biobank of a total of ten *BRAF*^*V600E*^ CRC PDOs. Models were extensively molecularly characterized as well as tested for response to the combination of erlotinib and encorafenib (E + E or anti-EGFR/BRAF therapy). Since cetuximab did not work in our culture system, we used erlotinib as anti-EGFRi. For the first time, we identified mechanisms of resistance as well as features of sensitivity to E + E that are similar to those mechanisms previously identified in data from clinical trials [21].

In our analysis of DNA mutations associated with response, we identified PTEN (both mutations and loss) and *PIK3R1* alterations to be mainly present in sensitive models. Using mass spectrometry, we also found that sensitive models had significantly higher levels of p-AKT at baseline as compared to resistant models and this was correlating with PTEN alterations. Together, these findings suggest that the PI3K/PTEN/p-AKT axis could be used as marker of response to E + E. Previously, van Geel et al. [13] reported that *BRAF*^*V600E*^ mCRC patients with *PIK3CA/PTEN* alterations had longer survival upon treatment with either cetuximab and encorafenib or cetuximab, encorafenib and alpelisib, suggesting that the above mentioned alterations might play a role in predicting longer responses to anti-EGFR/BRAF therapy. Kopetz et al. [21] did not report data regarding *PTEN* alterations but patients carrying a mutation in *PIK3CA* showed a smaller hazard ratio (HR) for OS in favour of anti-EGFR/BRAF therapy as compared to *PIK3CA* WT patients. Moreover in his analysis, Kopetz et al. [21] was mainly looking at correlation between mutations and response to either cetuximab + encorafenib or standard chemotherapy rather that looking if within the cetuximab + encorafenib arm the presence of *PIK3CA* mutation could help predict response. Although the prior results were obtained from a small phase I study [13] or lacked statistical significance/addressed the question in a different way [21], the three independent studies including our in vitro data show similar results, indicating the need to further investigate PIK3CA/PTEN/p-AKT alterations as biomarkers of response to anti-EGFR/BRAF therapy.

Elez et al. previously reported *RNF43* mutations as biomarker of response to anti-EGFR/BRAF therapy in *BRAF*^*V600E*^ MSS mCRC patients [40]. On the other hand, Kopetz et al. [21] could not confirm this observation. In line with Kopetz et al. [21], our analysis did not show an association between mutations in *RNF43* and response to E + E. The observed disparity of our data with the report of Elez et al. [40] could be driven by the fact that the vast majority of our models are dMMR and that all our models are nearly *RNF43* mutated thus rendering the study of such relationship difficult. Because of these incongruent results, further studies might be required to better understand the role of *RNF43* mutations in predicting response to anti-EGFR/BRAF therapy.

Further, when we examined the association of response to treatment with E + E with several gene expression signatures specific for CRC, namely the BRAF-like signature, the CMS and the BM classification, we failed to find any association. This finding is again in line with Kopetz et al. [21]. However, *BRAF*^*V600E*^ CRC falling into the BM1 classification have been reported to have high levels of p-AKT, KRAS/AKT and EMT markers [29] and have a better outcome under anti-EGFR/BRAF therapy compared to BM2 tumors [30]. Moreover, a potential role of EMT activation in response to cetuximab in RAS/BRAF wild-type mCRC has also been reported in two independent cohorts [17, 41]. As we found that p-AKT was more highly expressed in sensitive models, which is a feature of the BM1 subtype, we further investigated whether also EMT markers could be used as surrogates for response. While the expression of EMT genes was equally distributed among sensitive and resistant models, the sum of the tested EMT markers at protein level, in particular SMAD4, was slightly higher in sensitive models. Although the observed differences were not statistically significant, potentially because of the small sample cohort, our data might warrant further in vivo validation.

Analysis of gene expression after treatment showed a clear down-regulation of retinoblastoma, cell cycle, DNA damage and glycolysis pathways in sensitive models. More interestingly, directly comparing expression in resistant versus sensitive models, we observed an upregulation of glycolysis, mTORC, PIK3CA and ROS pathways upon treatment in resistant models. The increase of mTORC as well as PIK3CA pathways in resistant models was in line with our protein data that showed an increase of p-AKT upon treatment in resistant but not sensitive models. The observed increase of p-AKT might be related to the deregulation of the ROS pathway, which we also observed at gene expression and protein levels. Moreover, the extent of p-AKT increase in resistant models upon treatment might suggest the use of either a PIK3CAi oder a pan-AKTi in combination with E + E to reverse the intrinsic resistence.

Furthermore, we observed a significant downregulation of NOX5 upon treatment and a corresponding enrichment of the ROS pathway only in resistant models. We further used proteomics data to better understand if the enrichment observed was more towards an activation or downregulation of the pathway. We consistently found a downregulation of activators and an activation of inhibitors, thus indicating an inhibition of the ROS pathway upon treatment in resistant models. Kopetz et al. [21] reported a potential association between the SBS17 mutational signature and acquired resistance to anti-EGFR/BRAF therapy. This signature has been reported to be associated to ROS pathway [42] and ROS levels have been shown to be associated to primary resistance to BRAF inhibition in melanoma [43]. While our data point towards an inhibition of the ROS pathway upon treatment as marker of primary resistance, Kopetz et al. [21] did not specify the activation status of the pathway for the association between ROS and acquired resistance. However, taken together these data indicate that activation status of the ROS pathway might represent a biomarker of resistance to anti-EGFR/BRAF therapy warranting further inverstigation.

Along with the downregulation of the ROS pathway and the corresponding p-AKT increase we also observed an increase of anti-apoptotic proteins upon treatment that correlated with the survival of resistant models. Indeed, both the use of a PIK3CAi/pan-AKTi and a BCLi could increase cell death upon E + E in resistant models which warrants further validation in clinical setting.

We found histology to be one of the strongest clinical parameters associated with response to E + E. All our sensitive models were derived from well differentiated tumors while all resistant models from either poorly differentiated or mucinous tumors. When we investigated the cell of origin and cell composition of our models, we found that well differentiated models, i.e. sensitive models, were mainly enriched with enterocytes and Paneth cells while poorly differentiated, i.e. resistant models, were mainly enriched with neuroendocrine cells. Digiacomo et al. [44] reported high prevalence of neuroendocrine differentiation in *BRAF*^*V600E*^ mCRC while Fassan et al. [45] highlighted the negative impact of this subgroup in terms of PFS and OS. Information regarding histology or neuroendocrine differentiation and response to anti-EGFR/BRAF therapy in *BRAF*^*V600E*^ mCRC are lacking, currently preventing validation of our findings in clinical data. Nevertheless, we report a single case for which matching information of histology and scRNA cell subtyping was available, thus highlighting the need for further investigation.

## Conclusion

We show that *BRAF*^*V600E*^ CRC PDOs generated from a cohort of CRC patients at various disease stages can reproduce findings from completely independent clinical patient cohorts. In particular, we identified the PI3K/PTEN/p-AKT axis as potential biomarker of sensitivity and confirmed ROS as biomarker of resistance to anti-EGFR/BRAF therapy in the overall study cohort. These biomarkers warrant further prospective in vivo validation. If validated, they might help in the future the upfront selection of responders to anti-EGFR/BRAF therapy. Moreover, we suggest the combination of BCLi and PIK3CAi/pan-AKTi in intrinsically resistant patients to be tested. Finally, we suggest histology and cellular composition as important factors of resistance urging retrospective and prospective in vivo validation. A key limitation of our study is the small sample size of our PDO cohort, which may limit the ability to achieve statistical significance in some cases. This also hinders the ability to specifically assess whether the observed phenotypes differ between clinically relevant *BRAF*^*V600E*^ subgroups, such as MSI-H and MSS tumors. Additionally, the lack of in vivo data further represents a significant limitation. Nevertheless, the identification of similar features using different omics analyses and the external validation with prior clinical data partially helps overcome this limitation. Moreover, intrinsic limitations of PDOs are likely influencing the ability to find certain associations. For instance, the absence of a tumor microenvironment in our culture system may explain why we did not observe an association between the inflammatory pathway and treatment response, as reported by Kopetz et al. [21]. Additionally, this limitation prevents us from comparing drug responses between targeted agents like E + E and immunomodulators such as immunotherapy. The lack of autologous lymphocytes hampers this possibility and restricts our ability to differentiate between samples that could benefit from immunotherapy versus those that may respond better to E + E. To address these critical clinical questions, particularly for MSI-H *BRAF*^*V600E*^ mCRC patients, it is essential to develop future protocols that incorporate the tumor microenvironment, especially autologous lymphocytes. Such protocols would enhance the utility of PDOs in addressing clinical questions.

In conclusion, the limited size of our cohort can not provide statement regarding the different clinically relevant *BRAF*^*V600E*^ subgroups. Our data require future in vivo validation as well as a deeper understanding of the biological mechanisms underlying the observed association between phenotypes and biomarkers. Nevertheless, our results suggest that in future clinical trials patients receiving anti-EGFR/BRAF therapy beyond first line should be stratified based on alterations in the PI3K/PTEN/p-AKT axis and that non-responders should be treated with an additional PIK3CAi/pan-AKTi or BCL2i inhibitor. Finally, the results of our work support the use of PDOs for biomarker discovery. The use of PDOs should be considered early in drug development to help the identification of mechanisms of response and better select patients who might or not benefit from certain treatments.

## Supplementary Information

Supplementary Material 1.

Supplementary Material 2: Suppl. Figure 1. Correlations of Doubling Time, IC50 and AUC. a, Scatter plot showing strong correlation between IC50 and the AUC values of ten BRAF^V600E^ PDOs treated with increasing doses of erlotinib and encorafenib (r = 0.74, *p* < 0.05, Pearson). b, Scatterplot showing no significant correlation between IC50 values of ten BRAF^V600E^ PDOs treated with increasing doses of erlotinib and encorafenib and their doubling time (*r* = -0.12, p > 0.05, Spearman).c, Scatterplot showing no significant correlation between AUC values of ten BRAF^V600E^ PDOs treated with increasing doses of erlotinib and encorafenib and their doubling time (r = -0.24, p> 0.05, Spearman). The models HCT166 and HCT156 have identical AUC and similar doubling time, thus letting their points to overlap and not being clearly evident in our graph. This is the reason why the scatter plot contains only nice points even if all ten models were included.Suppl. Figure 2. Synergy Scores of erlotinib and encorafenib in PDOs. (a-f) ZIP synergy scores of ten BRAF^V600E^ PDOs treated with increasing doses of erlotinib or encorafenib. Values above 10 correspond to combinations that result in synergistic effects (red), values below -10 correspond to combination that result in an antagonistic effect (green) and values between 10 and -10 correspond to additivity. Suppl. Figure 3. Validation of mass spectrometry data. a, Western Blot of p-RSK2, p-P90, p-P70, p-AKT and p-ERK expression in ten BRAF^V600E^ CRC PDOs at baseline. Models are annotated as sensitive (green), resistant (purple) and intermediate (grey). GAPDH was used as loading control. Blots are representative of at the least two biological replicates. b, Western Blot of p-RSK2, p-P90, p-P70, p-AKT and p-ERK expression in three sensitive models upon E+E treatment (3.1 µM erlotinib + 2.9 µM encorafenib). GAPDH was used as loading control. Blots are representative of at the least two biological replicates. c, Western Blot of p-RSK2, p-P90, p-P70, p-AKT and p-ERK expression in two resistant models and one intermediate model upon E+E treatment (3.1µM erlotinib + 2.9 µM encorafenib). GAPDH was used as loading control. Blots are representative of at the least two biological replicates. Dose response curves of OT212, HCT161 and HCT156 treated with single agent alpelisib (d) or capivasertib (e) or the E+E with either a fixed dose of alpelisib (f-h) corresponding to the Cmax (HCT156 and HCT161= 5.4 µM) or corresponding to the IC30 (OT212= 0.7µM) or treated with a fixed dose of capivasertib corresponding to the Cmax (3.5 µM). For each drug concentration ATP levels were assessed, normalized to DMSO. Data were normalized, transformed in logarithms and fit into dose response curve. The graphs have a linear x-axis and are labeled with antilog. Each data point is the mean of at least three independent biological replicates and error bars represent the mean ± SEM. The median Z-factor score across all screening plates for E+E+alpelisib was 0,74(n = 54; upper and lower quartile = 0,86 and 0,54 respectively) while for E+E+ capivasertib was 0,77 (n = 54; upper and lower quartile = 0,86 and 0,61 respectively). The individual IC50 observed with their corresponding 95% CI are summarized for each model as follows: OT212: E+E IC50 3,9 µM (95% CI 0,7926 to 19,30), alpelisib IC50 2,6 µM (95% CI 1,677 to 4,160), capivasertib IC50 15,21 µM (95% CI 1,969 to 117,5), E+E+ alpelisib 0,07 µM IC50 (95% CI 0,02748 to 0,2273), E+E+capivasertib IC50 0,09 µM (95% CI 0,02770 to 0,2986); HCT161: E+E IC50 not applicable since the program could not generate an IC50 based on the fitting curve (95% CI, not applicable), alpelisib IC50 not applicable since the program could not generate an IC50 based on the fitting curve (95% CI, not applicable), capivasertib IC50 not applicable since the program could not generate an IC50 based on the fitting curve (95% CI, not applicable), E+E+ alpelisib IC50 21,7 µM (95% CI 5,194 to 90,76), E+E+capivasertib IC50 0,5 µM (95% CI 0,1036 to 2,632); HCT156: E+E IC50 5,7 µM (95% CI 0,9404 to 34,70), alpelisib IC50 132,7 µM (95% CI 0,06395 to 275210), capivasertib IC50 12,9 µM (95% CI 7,878 to 21,11), E+E+ alpelisib IC50 0,10 µM (95% CI 0,01804 to 0,5964), E+E+capivasertib IC50 0,22 µM (95% CI 0,09225 to 0,5700). Suppl. Figure 4. Multi-omic signatures associated with E+E response. a, Heatmap (left) showing unsupervised clustering-based baseline gene expression. Data were normalized using z-score transformation. Heatmap (right) displaying unsupervised clustering based on gene expression after treatment with E+E (3.1 µM erlotinib + 2.9 µM encorafenib). MSI/MMR status,BRAF mutation, classification of resistant, sensitive and intermediate models, as well histology is annotated above. b, Principal component analysis (PC) correlated with clinically relevant information and response data. Asterix display significant positive and negative correlation c, Heatmap of gene set variation analysis (GSVA) of normalized enrichment score in bulk RNA sequencing of tenBRAF^V600E^ CRC PDOs. Gene signatures are obtained from Sehgal et al. [32]. d, Representative Western Blot of E-cadherin, N-cadherin, SMAD4, total and phosphor SMAD2 and Vimentin expression in ten BRAF^V600E^ CRC PDOs at baseline. Models are annotated as sensitive (green), resistant (purple) and intermediate (grey). GAPDH was used as loading control. Suppl. Figure 5. EMT and ROS as associated with E+E response. a, Stacked bar chart displays expression of SMAD4, phosphor and total SMAD2 in ten BRAFV600E CRC PDOs relative to control determined by Western Blot, refers to Suppl Fig 5D b, Heatmap of normalized enrichment scores of pathways from Hallmark gene sets from Human MSigDB Collections [46] at baseline using single sample Gene Set Enrichment Analysis (ssGSEA) in ten BRAFV600E CRC. c, Heatmap of normalized enrichment scores pathways from Hallmark gene sets from Human MSigDB Collections [46] using ssGSEA in three sensitive, two resistant and one intermediate BRAFV600E CRC PDO model. For post-treatment analysis, log2 fold change values of treated samples with erlotinib and encorafenib (Cmax) versus the control (DMSO) were used to calculate NES via ssGSEA. d, Heatmap shows the log2 fold change of cell stress and apoptosis pathway proteins of three sensitive models, two resistant models, and one intermediate model upon treatment with E+E (3.1 µM erlotinib + 2.9 µM encorafenib) versus the control (DMSO). The data were determined by Proteome Profiler Human Cell Stress Arrays and Proteome Profiler Human Apoptosis Arrays. Data represent one biological replicate. Suppl. Figure 6. Deciphering the role ROS pathway and related proteins with treatment response. a, Cartoon is illustrating the ROS pathway in resistant models upon treatment with E+E. Genes and proteins that are upregulated or downregulated in resistant versus sensitive models and after treatment with erlotinib and encorafenib are colored in purple or orange, respectively. b-e, Dose response curves of OT212, HCT161 and HCT156 treated with single agent obatoclax. Model OT212 (C), HCT161 (D), and HCT156 (E) were treated with the dual combination of E+E and the triple combination of E+E+fixed dose of obatoclax corresponding to the IC30 of each model (HCT156 and OT212=0.08 µM; HCT161=2 µM). For each drug concentration ATP levels were assessed, normalized to DMSO. Data were normalized, transformed in logarithms and fit into dose response curve. The graphs have a linear x-axis and are labeled with antilog. Each data point is the mean of at least two independent biological replicates and error bars represent the mean ± SEM. The median Z-factor score across all screening plates was 0,82(n = 49 ; upper and lower quartile = 0,87 and 0,62 respectively). The individual IC50 observed with their corresponding 95% CI are summarized for each model as follows: OT212: E+E IC50 3,9 µM (95% CI 0,7926 to 19,30), obatoclax IC50 0,17 µM (95% CI 0,1221 to 0,2532), E+E+ obatoclax IC50 0,3 µM (95% CI 0,05499 to 2,861); HCT161: E+E IC50 not applicable since the program could not generate an IC50 based on the fitting curve (95% CI not applicable), obatoclax IC50 3,35 µM (95% CI 2,676 to 4,195), E+E+ obatoclax IC50 not applicable since the program could not generate an IC50 based on the fitting curve (95% CI not applicable); HCT156: E+E IC50 5,7 µM (95% CI 0,9404 to 34,70), obatoclax IC50 0,17 µM (95% CI 0,06498 to 0,4968), E+E+ obatoclax IC50 0,03 µM (95% CI 0,008832 to 0,1467). Suppl. Figure 7. Correlation of treatment response with intestinal cell types. Boxplots showing the mean module score of intestinal cell types in resistant (purple) vs. sensitive (green) BRAFV600E CRC PDO models. Gene lists for module score calculations were obtained from PanglaoDB [36], representing cell-type-specific gene sets. Module scores were calculated using the Seurat function AddModuleScore.

Supplementary Material 3. Supplementary tables

## Data Availability

The datasets used and/or analysed during the current study are available from the corresponding author on reasonable request.

## Abbreviations

5-FU: 5-Fluorouracil
Anti-EGFRi: Anti-EGFR inhibitor
AUC: Area under the curve
BCLi: BCL2 inhibitor
BM: BRAF mutant
BRAFi: BRAF inhibitor
CIN: Chromosomal instability
CMS: Consensus Molecular Subtypes
CRC: Colorectal cancer
dMMR: Deficient mismatch repair
EGFR: Epidermal growth factor receptor
EMT: Epithelial-mesenchymal transition
ESMO: European Society of Medical Oncology
FDR: False Discovery Rate
GSVA: Gene set variation analysis
H&E: Hematoxylin and Eosin
HR: Hazard ratio
IHC: Immunohistochemistry
LC–MS/MS: Liquid chromatography-tandem mass spectrometry
mCRC: Metastatic colorectal cancer
MEKi: MEK inhibitors
MSI: Microsatellite instability
MSI-H: Microsatellite instability high
MSS: Microsatellite stable
NCCN: National Cancer Network
OS: Overall survival
PAF: Paraformaldehyde
pan-AKTi: Pan-AKT inhibitor
PCA: Principal Component Analysis
PDOs: Patients derived organoids
PFS: Progression-free survival
PI3Ki: PI3K inhibitors
RTK: Receptor Tyrosine Kinases
ssGSEA: Single sample Gene Set Enrichment Analysis
SVM: Support vector machine
WES: Whole exome sequencing
