# Supplementary material for: BRAFV600E patient derived colon cancer organoids identify biomarkers of response to EGFR and BRAF inhibition and replicate clinical data
Source: J Exp Clin Cancer Res. 2026 Mar 25;45:92. doi: 10.1186/s13046-026-03699-2 (PMC13063721; doi:10.1186/s13046-026-03699-2)
Supplement: Supplementary file 1 — Supplementary Material 1. [file 13046_2026_3699_MOESM1_ESM.docx]

**Supplementary Material**

**Materials and Methods**

1. **Specimen procurement**

In total, ten patient-derived organoids (PDOs) from patients with colorectal cancer were used for this study. Six out of ten PDOs were established from chemo naïve tissue and obtained from a collaboration with the Japanese Cancer Center upon MTA. Two models were obtained from the OncoTrack preclinical platform [17 of main manuscript]. Finally, two patients were recruited at the Charite Universitätsmedizin Berlin Mitte and tissues we obtained from their metastatic sites and processed as reported below in paragraph 2. In all cases, patients signed informed consent after ethical committees approved the study protocols. Patient characteristics including gender, MMR status, tumor location, origin of the cohort, tumor stage and sites of metastasis can be found in table 1.

1. **Tumor dissociation, organoid derivation and culture**

Fresh tumor tissue obtained from a bronchial lymph node biopsy of a 31-year old male patient and fresh tumor tissue from a liver resection specimen of a 58-year old female patient, both diagnosed with metastatic *BRAF^V600E^* CRC, were submitted to our laboratory to generate the two organoid models ICP_CRC_01 and M2. Tissue was collected in P/S medium (Advanced DMEM/F12 (Gibco, 12634028) containing 1x Penicilin-Streptomycin (Thermo Fisher Scientifics, 15140122), 10 mM HEPES (Thermo Fisher Scientifics, 15630056)) with 5 µM Y27632 2HCI Rock inhibitor (AbMole, M1817)) and 1% gentamicin (Thermo Fisher, 15750060). For the first week, complete medium was supplemented with 1.25 µg/ml Amphotericin B (Sigma) and 10 µM Rock Inhibitor. Afterwards ICP_CRC_01 was grown in complete medium and M2 in complete medium supplemented with 500 nM A 83-01 (Tocris, 2939). All organoids were further established and cultured following the Oncotrack protocol [17]. Single cells were cultivated in complete medium containing Advanced DMEM/F12, 1x Penicilin-Streptomycin, 10 mM HEPES, GlutaMAX Supplement (Thermo Fisher Scientific, 35050061), N-Acetyl-L-Cysteine (Sigma Aldrich, A9165), N2-Supplement (Gibco, 17502001), B27-Supplement (Gibco, 12587001), human bFGF (Peprotech, AF-100-18B) and human EGF (Sigma Aldrich, E9644). The HCT166 models was the only one cultures in presence of WNT conditional medium. Cultures were passaged when they reached a diameter of approximately 400-600 µm and 80-90% confluency. Organoids were closely monitored and regularly tested for mycoplasma contaminations. Low passage numbers and highly proliferative organoids were frozen. Organoids from 3-4 wells were collected and resuspended in Recovery^TM^ cell culture freezing medium (Thermo Fisher Scientific, 12648010) per cryotube.

1. **Immunohistochemistry**
   1. **Embedding of Organoids for Immunohistochemistry**

Organoids were expanded to a minimum of 18 wells of a 24-well plate and cultivated till they reached a size of 200 μm and 70-80% confluency. Organoids were collected, gently washed with ice cold PBS (Thermo Fisher Scientific, 14190169) and incubated on ice for 60 min in pre-chilled Cell recovery solution (Corning, 354253). Pellets were washed two times with ice cold PBS and afterwards fixed with 4% paraformaldehyde (PAF) (Roth, 4979) for one hour at room temperature. Organoids were again washed two times with PBS and finally resuspended in pre-warmed HistoGel^TM^ (Thermo Fisher Scientific, 12006679) and embedded in cryomolds for H&E and dMMR protein staining.

- 1. **Hematoxylin-Eosin (HE) Staining**

Serial 3 µm paraffin sections of the organoids were mounted on glass slides and deparaffinized and rehydrated using a standard protocol. HE staining was performed by incubating slides in hematoxylin for 8 minutes, followed by bluing in tap water for 8 minutes. Sections were then stained with eosin for 2.5 minutes, dehydrated, and mounted using a Sakura automated coverslipper.

- 1. **Immunohistochemical Staining of Mismatch Repair (MMR) Proteins**

Immunohistochemical analysis of MMR proteins (MLH1, MSH2, MSH6, and PMS2) was performed on 3 µm paraffin sections using an automated staining system (Ventana BenchMark Ultra, Roche). Antigen retrieval was conducted via heat-induced epitope retrieval using CC1 buffer (pH 9) for all MMR proteins. The primary antibodies, all from Roche Ventana, were applied as follows: MLH1 (Clone M1), MSH2 (Clone G219-1129), MSH6 (Clone SP93), and PMS2 (Clone A16-4). Detection was performed using the OptiView DAB Detection Kit. Sections were counterstained with hematoxylin, dehydrated, and mounted.

- 1. **Evaluation of Stainings**

HE staining was used for morphological assessment of the organoids. Immunohistochemistry was evaluated by an experienced pathologist. Loss of MMR protein expression was defined as the complete absence of nuclear staining in tumor cells, with surrounding stromal tissue serving as an internal positive control. The results were classified according to the presence of dMMR or pMMR.

1. **Short term proliferation assays**

Short term proliferation assays were performed according to the Oncotrack protocol [17]. Upon reaching 70-80% confluency in a 24 well plate, PDOs were collected and digested with TrypLE^TM^ Express to a single cell level. Cells were dyed with Acridine Orange/Propidium Iodide (Logos Biosystems, F23001) and counted with Luna Counter. 2,000-4,000 cells were plated with 6 µl matrigel drops in three technical triplicates in flat clear bottom black 96-well plates (Corning) in presence of medium, whose composition is reported above. Single cells were grown for four days at 37°C, and then treated with either increasing doses of single agents erlotinib (Selleckchem, S7786), encorafenib (Selleckchem, S7108), alpelisib (Selleckchem, S2814), capivasertib (MedchemExpress, HY-15431), obatoclax (Selleckchem, S6709), and their respective dual and triple combinations. Cells were treated with seven increasing concentrations that ranged from 0.00001 μM-10 μM for erlotinib and encorafenib. For the dual combination of erlotinib and encorafenib drugs were diluted in a 1:1 constant drug ratio ranging from 0.00001 μM to 10 μM. For the triple combinations we used five increasing doses of erlotinib and encorafenib (0.001 µM – 10 µM) in a 1:1 ratio and added either a fixed dose of alpelisib, capivasertib or obatoclax. Fixed dose of alpelisib corresponded either to its Cmax or the IC30 (HCT156 and HCT161= 5.4 µM and OT212=0.7 µM). Fixed dose of capivasertib, corresponded to its Cmax (3.5 µM) and fixed dose of obatoclax corresponded to the IC30 of each model (HCT156 and OT212=0.08 µM; HCT161=2 µM). As negative control medium containing DMSO was used. As positive control 3 μM staurosporine (Selleckchem, S1421) was included. After 72 hours of incubation with the above-mentioned drugs, ATP levels were assessed using a 1:1 ratio of Cell Titer Glo Luminescent Cell Viability Assay (Promega, G7571). To ensure proper cell lysis, plates were agitated on a plate shaker for 20 min and additionally incubated for 10 min at room temperature, following the Promega protocol.

1. **Dose response curves and area under the curve**

For data analysis and visualization GraphPad Prism (version 9, GraphPad, San Diego, CA, USA) was used. For each drug test the mean luminescent signals (RLU) of three technical replicates were used. Biological replicates were normalized to luminescent signals of the negative controls (i.e medium containing DMSO) and logarithmically transformed using X=log(X). To generate dose response curves the nonlinear regression curve fit method (log(inhibitor) vs. normalized response-- variable slope) was applied. The dose response curves represent the mean of at least two biological replicates with relative standard error of mean (SEM) for each concentration.

IC50 values were retrieved from curve fitting in GraphPad Prism. AUC was calculated in R and cut offs were determined using the Jenks Natural Breaks in Excel.

Moreover, a statistical measure of assay quality was determined by calculating the Z-factor as follows:

Z-factor was calculated as the following equation adapted from Du et al. [1]:

$$Z=1-({3SD}_{control}+{3SD}_{Blank} )/({RLU}_{control} -{RLU}_{Blank})$$

SD_control_ and SD_Blank_ are the standard deviations and RLU_control_  and RLU_Blank_ are the average luminescent signals (RLU) of three technical replicates of each plate of negative control (DMSO or medium) and Blank wells without organoids. A z-factor can be considered as robust if it is between 0.5 and 1 [2]. However, for organoid cultures the minimum can also be considered as 0.4 based on the technical complexity of organoids [3]

**Statistics and reproducibility.** Short term proliferation assays were performed in three technical and at least two biological replicates. Only for the drug tests where E+E is compared to triple combinations using fixed doses of alpelisib, capivasertib or obatoclax, all biological replicates of E+E from all experiments were considered (n=12). Microscopic images, and images from Immunohistochemistry of PDOs are representative for the organoids. Statistical tests are indicated in figure legends or in the specific Material and Methods sections.”

1. **Mutational profiling**
   1. **DNA isolation and sequencing**

DNA analysis was performed on PDOs at basal level. For this, Organoids were plated in absence of drugs into 12-18 wells of a 24-well plate with a 40-50% confluency. They were cultivated in presence of regular medium and harvested when they reached 80% confluency and at least the size of >200 μm. DNA was isolated using the DNeasy Blood & Tissue Kit (Qiagen, 69504) and following manufacturer instructions. Concentration was measured by using Qubit dsDNA High Sensitivity Assay Kit (Thermo Fisher Scientific, Q32851). Moreover, DNA integrity was assessed by TapeStation. Whole exome sequencing was performed on an Illumina NovaSeq 6,000 with 150 base pairs end.

- 1. **Data preparation**

**Alignment of WES data**

For mapping reference genome GRCh38.d1.vd1 was used, which contains virus sequences and decoy to enable EBV and HPV detection. Alignment was done using bwa (version 0.7.17) and the corresponding statistics using samtools stats (version 1.9+htslib-1.9). Pile-up for selected oncogenes was done with bcftools (version 1.16). Visualization of variant allele frequencies was prepared using R (version 4.2.2) with packages vcfR (version 1.13.0), tidyverse (version 1.3.2), readxl (version 1.4.1) and ggpubr (version 0.5.0).

**Whole-Exome Sequencing (WES) Mutation Analysis**

Mutations were identified using the MuTect2 pipeline (GATK version 4.1.4.1) [4] with default parameters in tumor-only mode. Variants were filtered against the COSMIC database for CosmicCodingMuts and retained if their allele frequency (AF) exceeded 0.25. Final mutation calls were selected through a stringent filtering process and functionally annotated by ANNOVAR using Ensembl (v.93) [5], COSMIC (v.98) [6], ClinVar (201706) [7], gnomAD (r.2.0.1) [8] and dbSNP (v.150) [9].

**Mutational Exclusivity Analysis**

Mutational exclusivity was analyzed by binarizing mutations (mutated = 1; not mutated = 0) and performing Fisher’s Exact Test to compare resistant versus sensitive samples. Results were filtered for significance using p-values corrected for multiple testing using the Benjamini-Hochberg method form stats R package (v 4.5.0) (p.adj < 0.05). Intermediate models were excluded from this analysis but still represented in the graph for completeness and comparison.

1. **Gene expression profiling**
   1. **RNA isolation and sequencing**

For RNA analysis at basal level all organoid models were plated into 12-18 wells of a 24-well plate. They were cultivated and harvested when they reached 80% confluency and at least the size of >200 μm. For gene expression analysis after drug perturbation, organoid models were plated for untreated and treated conditions and cultivated for four days. Organoids were then treated with fixed doses of erlotinib and encorafenib, which corresponds to the individual Cmax of the drugs. Organoids were then collected after 24 hours after treatment. Total RNA extraction was performed using the RNeasy Micro Kit (Qiagen, 74004) following the manufacturer instructions. RNA concentration was measured using Qubit RNA High Sensitivity Assay Kit (Thermo Fisher Scientific, Q32852). Samples with low 260/280 ratio were purified by using the RNA Clean& Concentrator-5 Kit^TM^ (Zymo Research, R1015). RNA integrity was evaluated by TapeStation. Sequencing was performed on a NovaSeq 6,000 with 150 base pairs paired end.

- 1. **Data preparation**

**RNAseq Alignment and read counts**

RNA sequencing data was aligned to reference genome GRCh38 (patch 7, chromosomes and scaffolds) using STAR (v 2.7.3a). using the following parameters: --outSAMunmapped Within --outFilterType BySJout –outFilterMultimapNmax 20 --alignSJoverhangMin 8 --alignSJDBoverhangMin 1 --outFilterMismatchNmax 999 --outFilterMismatchNoverLmax 0.04 --alignIntronMin 20 --alignIntronMax 1,000,000 --alignMatesGapMax 1,000,000 Reads were assigned to genes with featureCounts (from the Bioconductor package rsubread v 1.34.0) using Gencode annotations (v 25, GRCh38 p7, chromosomes and scaffolds). Later downstream analysis was performed using R version 3.4.3,3.6.0 and 4.2.2 and python version 3.9.12 Technical replicates were collapsed using the collapseReplicates function from the DESeq2 package [10]. Count data was normalized and transformed into regularized log (rlog) values using the DESeq2 package.

**Principal Component Analysis (PCA)**

Principal Component Analysis (PCA) using the PCA module from the sklearn.decomposition package in Python's scikit-learn library (1.0.2) was conducted on rlog-transformed basal expression data, focusing on the top 2,000 most variable genes (Genes were ranked based on their variance, and the top 2,000 most variable genes were selected for downstream analysis). The resulting principal components (PCs) were associated with clinical annotation data. Spearman correlation was employed for continuous variables, while the Wilcoxon rank-sum test was used for categorical variables with two groups. For categorical variables with more than two groups, the Kruskal-Wallis test was applied.

**Differential Expression Analysis**

Differential expression analysis between resistant and sensitive groups under basal conditions was conducted using DESeq2 (1.42.0). To examine the effects of gene expression changes following treatment, genes in the sensitive and resistant groups were independently identified using DESeq2 by comparing post-treatment expression to untreated conditions. Of the significantly differential expressed genes (adjusted p-value < 0.05) we identified those common and unique to sensitive and resistance group using intersect analysis. Additionally, significant changes showing opposite directions of regulation between the two groups were identified using the same approach.

**CMS Classification**

CMS classification was conducted using the CMScaller package 0.99.2 [11] on both basal and post-treatment RNASeq data. CMScaller utilizes a nearest template prediction method to classify each sample into one of the CMS subtypes (CMS1–CMS4) based on a calculated prediction distance and its corresponding p-value. Samples with p-values greater than 0.05 are considered non-significant and remain unclassified. Furthermore, CMS template genes serve as input for ssGSEA scoring, which evaluates the level of enrichment of CMS-specific signatures within the samples.

**BM Classification**

The TCGA dataset (n= 218) [12], annotated with BM classification, was merged with the study dataset. To account for technical variability and systematic batch effects that could arise from combining datasets generated under different conditions, the ComBat algorithm from the sva package (3.46.0) [13] was used. Subsequently, a support vector machine (SVM) classifier from the sklearn package (1.0.2) [14] was utilized for sample classification from study dataset into the BM1 and BM2 subtypes based on reported genes significantly associated with BM subtype annotation [15]. The model was first evaluated using 5-fold cross-validation on the training dataset to assess its performance and variability across different folds.

**Single-Sample Gene Set Enrichment Analysis (ssGSEA)**

Gene set enrichment analysis was conducted using the GSVA (Gene Set Variation Analysis) [16] package on Hallmark gene sets [17] to evaluate pathway activity.

1. **Single cell sequencing**
   1. **Library preparation and sequencing**

Single cell RNA sequencing analysis was performed at basal level. For this, organoid models were plated into 24-wells, cultured with medium and harvested after 7 days and dissociated into single cells using TrypLE^TM^ Express. Cells were further filtered through a 20 µm strainer counted and resuspended in PBS + 0.01% BSA. Single cells were fixed using the Chromium Next GEM Single Cell Fixed RNA Sample Preparation Kit (10x Genomics, PN-1000414) and libraries were prepared using the Chromium Fixed RNA Kit,Human Transcriptome (10x Genomics, PN-1000475) according to manufacturers instructions. Library quality was determined by TapeStation. Sequencing was performed on NovaSeq X Plus (Illumina) (1600 mio. reads per library).

- 1. **Single-cell analysis**

The initial processing of raw sequencing data was carried out using CellRanger v7.2.0 multi (10x Genomics), with transcript alignment performed against the 10x Genomics human genome reference (GRCh38-2020-A). Quality control and downstream analyses were conducted using Seurat v5.1.0. To ensure data quality, cells with fewer than 200 detected genes or genes present in fewer than three cells were excluded. Additionally, cells exhibiting more than 100,000 total reads or a mitochondrial transcript fraction exceeding 15% were removed to prevent potential artifacts. Gene expression counts were log-normalized using a scale factor of 10,000, and the 2,000 most highly variable genes were selected through the FindVariableFeatures function in Seurat. Finally, the normalized dataset was further processed by applying the ScaleData function to standardize gene expression values. To integrate multiple PDO samples, the IntegrateLayers function was applied using the CCAIntegration method. Furthermore, cell cycle effects were assessed using the CellCycleScoring function. Markers for resistant and sensitive samples were identified using the FindMarkers function. Potential cell of origin was assessed by using the AddModuleScore function with gene lists from PanglaoDB [18].The cell type with the highest module score for each cell was then identified. The proportion of each cell type per sample and class (sensitive/resistant) was calculated by determining the number of cells assigned to each cell type and dividing it by the total cell count in the sample. This was done separately for each sample and class.

1. **Proteomics sample process, LC-MS measurements and data analysis**

For protein analysis at basal level, organoids were plated into 12-18 wells of a 24-well plate with a 40-50% confluency. They were cultivated and harvested when they reached 80% confluency and at least the size of >200μm. Frozen pellets were lysed in a lysis buffer containing 8 M Urea, 50 mM Tris pH 8, 75 mM NaCl, 1 M EDTA, and protease and phosphatase inhibitors. Protein concentration was measured using the BCA assay. The model HCT178 was not included in the analysis because of the low amount of proteins in the sample analyzed, whereas the model ICP_CRC_01 was not included because it was not yet established at the time of the analysis. Therefore, a total of eight models were included in the final analysis. Samples were reduced with DTT, alkylated with IAA, and digested with Lys-C and Trypsin. Peptide samples were desalted using a C18 SPE cartridge and resuspended in a sample buffer. Label-free samples were measured before TMT labeling for quality control purposes. All analyzed models passed the quality controls, with no contamination with Matrigel proteins detected (data not shown, available upon request). Measurements were done on an Orbitrap Exploris 480 in data independent analysis (DIA) mode and data is analyzed using direct-DIA workflow in SpectroNaut 16. TMT labeling was performed using TMT16 reagents according to the manufacturer's instructions. Labeled samples were pooled and separated by high-pH reversed-phase liquid chromatography. Fractions were reduced to 28 fractions for global proteome and to 14 fractions for phosphopeptide enrichment by IMAC. Proteome and phosphoproteome data were acquired using Orbitrap Q-Exactive HFX instruments. The raw data was analyzed using MaxQuant (version 1.6.7.0) [19]. For pathway analysis, normalized reporter ion intensities of the phosphosites were used as input for single sample Gene Set Enrichment Analysis (ssGSEA) matching against PTMsigDB [20].

1. **Western Blot analysis**

Organoids were plated into 12-18 wells of a 24-well plate. They were cultivated and harvested when they reached 80% confluency and at least the size of >200μm for baseline analysis. For Western Blot analysis after drug perturbation, 12,000-24,000 cells/well were plated and then cultured for four days. On the fifth day, cells were treated with fixed doses of erlotinib and encorafenib (Cmax of each) and the pellet was collected after 24 hours after the treatment. Upon collection, samples were lysed with RIPA buffer supplemented with protease Inhibitor cocktail (Thermo Fisher Scientific) and Pierce Phosphatase inhibitor mini tablet (Thermo Fisher Scientific). Protein concentration was determined by BCA Protein Assay Kit (Thermo Fisher Scientific). Lysates were mixed with Bolt LDS sample buffer (Thermo Fisher Scientific), Bolt sample reducing agent (Thermo Fisher Scientific) and denaturized at 95°C for 10 min. 12.5 µg of protein per sample was loaded and separated by Bolt 4-12% Bis-Tris-Plus Gel (Thermo Fisher Scientific) in MOPS running buffer and transferred to a PVDF membrane. Antibodies against p-AKT (Ser473) (Cell Signaling, 9271), p-ERK (Thr202/Tyr204) (Cell Signaling, 4370), PTEN (Cell Signaling, 9559), p-RSK2 (Ser227) (Cell Signaling, 3556S), p-p90RSK (Ser380) (Cell Signaling, 12032), p-p70S6K (Ser371) (Cell Signaling, 9208), E-Cadherin (Cell Signaling, 3195), N-Cadherin (Cell Signaling, 13116), vimentin (Cell Signaling, 5741), SMAD4 (Cell Signaling, 46535), p-SMAD2 (Ser465/467) (Cell Signaling, 3108), SMAD2 (Cell Signaling, 3122), were used in 1:1,000 dilution in 5% BSA in TBS-T and GAPDH (Cell Signaling, 5174) was used as loading control in 1:10,000 dilution. Secondary antibodies Goat Anti- Rabbit IgG (H+L)-HRP Conjugate (Bio-Rad, 1706515) and Goat Anti- Mouse IgG (H+L)-HRP Conjugate (Bio-Rad, 1706516) were used in 1:10,000 dilution. Bands were visualized with Clarity and Clarity Max Western ECL Substrates (Bio-Rad, 1705060 and 1705062). Western Blot was imaged by ChemiDoc MP Imaging system and quantified by Bio-Rad Image Lab software.

1. **Synergy score**

The ZIP Synergy Score was calculated and visualized for the normalized datasets using the synergyfinder R package.

1. **Proteome Profiler**

Organoids were plated into 12-18 wells of a 24-well plate. They were cultivated and harvested when they reached 80% confluency and at least the size of >200μm for baseline analysis. For Western Blot analysis after drug perturbation, 12,000-24,000 cells/well were plates and then cultured for four days. On the fifth day, cells were treated with fixed doses of erlotinib and encorafenib (Cmax of each) and the pellet was collected after 24 hours after the treatment. Proteome Profiler Human Cell Stress Array Kit (R&D Systems, ARY018) and Proteome Profiler Human Apoptosis Array Kit (R&D Systems, ARY009) were used according to manufacturer instructions. Immunoreactive proteins were visualized by Clarity and Clarity Max Western ECL Substrates. Arrays were imaged by ChemiDoc MP Imaging system and quantified by Bio-Rad Image Lab software.

**Additional References**

[1] [Yuhong D.](https://pubmed.ncbi.nlm.nih.gov/?term=Du+Y&cauthor_id=32678871), [Xingnan L](https://pubmed.ncbi.nlm.nih.gov/?term=Li+X&cauthor_id=32678871)., [Qiankun N.](https://pubmed.ncbi.nlm.nih.gov/?term=Niu+Q&cauthor_id=32678871), [Xiulei M.](https://pubmed.ncbi.nlm.nih.gov/?term=Mo+X&cauthor_id=32678871), [Min Q.](https://pubmed.ncbi.nlm.nih.gov/?term=Qui+M&cauthor_id=32678871), [Tingxuan M.](https://pubmed.ncbi.nlm.nih.gov/?term=Ma+T&cauthor_id=32678871), [Calvin J K.](https://pubmed.ncbi.nlm.nih.gov/?term=Kuo+CJ&cauthor_id=32678871), [Haian F.](https://pubmed.ncbi.nlm.nih.gov/?term=Fu+H&cauthor_id=32678871) ^“^Development of a miniaturized 3D organoid culture platform for ultra-high-throughput screening” J Mol Cell Biol, 2020 Aug 1, 12(8):630-643. doi: 10.1093/jmcb/mjaa036.

[2] [Zhang](https://pubmed.ncbi.nlm.nih.gov/?term=Zhang+JH&cauthor_id=10838414) JH., [Chung](https://pubmed.ncbi.nlm.nih.gov/?term=Chung+TD&cauthor_id=10838414) TD., [Oldenburg](https://pubmed.ncbi.nlm.nih.gov/?term=Oldenburg+KR&cauthor_id=10838414) KR. „A Simple Statistical Parameter for Use in Evaluation and Validation of High Throughput Screening Assays” J Biomol Screen 1999;4(2):67-73.  doi: 10.1177/108705719900400206.

[3] [Francies](https://link.springer.com/protocol/10.1007/7651_2016_10#auth-Hayley_E_-Francies) AE., [Barthorpe](https://link.springer.com/protocol/10.1007/7651_2016_10#auth-Andrew-Barthorpe) A., [McLaren-Douglas](https://link.springer.com/protocol/10.1007/7651_2016_10#auth-Anne-McLaren_Douglas) A., [Barendt](https://link.springer.com/protocol/10.1007/7651_2016_10#auth-William_J_-Barendt) WJ & [Garnett](https://link.springer.com/protocol/10.1007/7651_2016_10#auth-Mathew_J_-Garnett) MJ. „ Drug sensitivity Assays of human cancer organoids cultures” Methods in Molecular Biology, pp 339-351, <https://doi.org/10.1007/7651_2016_10>).

[4] D. Benjamin, T. Sato, K. Cibulskis, G. Getz, C. Stewart, and L. Lichtenstein, “Calling Somatic SNVs and Indels with Mutect2,” Dec. 02, 2019. doi: 10.1101/861054.

[5] T. Hubbard, “The Ensembl genome database project,” *Nucleic Acids Res*, vol. 30, no. 1, pp. 38–41, Jan. 2002, doi: 10.1093/nar/30.1.38.

[6] J. G. Tate *et al.*, “COSMIC: the Catalogue Of Somatic Mutations In Cancer,” *Nucleic Acids Res*, vol. 47, no. D1, pp. D941–D947, Jan. 2019, doi: 10.1093/nar/gky1015.

[7] M. J. Landrum *et al.*, “ClinVar: improving access to variant interpretations and supporting evidence,” *Nucleic Acids Res*, vol. 46, no. D1, pp. D1062–D1067, Jan. 2018, doi: 10.1093/nar/gkx1153.

[8] M. Lek *et al.*, “Analysis of protein-coding genetic variation in 60,706 humans,” *Nature*, vol. 536, no. 7616, pp. 285–291, Aug. 2016, doi: 10.1038/nature19057.

[9] S. T. Sherry, “dbSNP: the NCBI database of genetic variation,” *Nucleic Acids Res*, vol. 29, no. 1, pp. 308–311, Jan. 2001, doi: 10.1093/nar/29.1.308.

[10] M. I. Love, W. Huber, and S. Anders, “Moderated estimation of fold change and dispersion for RNA-seq data with DESeq2,” *Genome Biol*, vol. 15, no. 12, p. 550, Dec. 2014, doi: 10.1186/s13059-014-0550-8.

[11] P. W. Eide, J. Bruun, R. A. Lothe, and A. Sveen, “CMScaller: an R package for consensus molecular subtyping of colorectal cancer pre-clinical models,” *Sci Rep*, vol. 7, no. 1, p. 16618, Nov. 2017, doi: 10.1038/s41598-017-16747-x.

[12] The Cancer Genome Atlas Network, “Comprehensive molecular characterization of human colon and rectal cancer,” *Nature*, vol. 487, no. 7407, pp. 330–337, Jul. 2012, doi: 10.1038/nature11252.

[13] Leek JT *et al.*, “sva: Surrogate Variable Analysis, R package version 3.56.0.”

[14] F. Pedregosa FABIANPEDREGOSA *et al.*, “Scikit-learn: Machine Learning in Python Gaël Varoquaux Bertrand Thirion Vincent Dubourg Alexandre Passos PEDREGOSA, VAROQUAUX, GRAMFORT ET AL. Matthieu Perrot,” 2011. [Online]. Available: http://scikit-learn.sourceforge.net.

[15] D. Barras *et al.*, “BRAF V600E Mutant Colorectal Cancer Subtypes Based on Gene Expression.,” *Clin Cancer Res*, vol. 23, no. 1, pp. 104–115, Jan. 2017, doi: 10.1158/1078-0432.CCR-16-0140.

[16] S. Hänzelmann, R. Castelo, and J. Guinney, “GSVA: gene set variation analysis for microarray and RNA-Seq data,” *BMC Bioinformatics*, vol. 14, no. 1, p. 7, Dec. 2013, doi: 10.1186/1471-2105-14-7.

[17] A. Liberzon, C. Birger, H. Thorvaldsdóttir, M. Ghandi, J. P. Mesirov, and P. Tamayo, “The Molecular Signatures Database Hallmark Gene Set Collection,” *Cell Syst*, vol. 1, no. 6, pp. 417–425, Dec. 2015, doi: 10.1016/j.cels.2015.12.004.

[18] O. Franzén, L.-M. Gan, and J. L. M. Björkegren, “PanglaoDB: a web server for exploration of mouse and human single-cell RNA sequencing data,” *Database*, vol. 2019, Jan. 2019, doi: 10.1093/database/baz046.

[19] J. Cox and M. Mann, “MaxQuant enables high peptide identification rates, individualized p.p.b.-range mass accuracies and proteome-wide protein quantification,” *Nat Biotechnol*, vol. 26, no. 12, pp. 1367–1372, Dec. 2008, doi: 10.1038/nbt.1511.

[20] K. Krug *et al.*, “A Curated Resource for Phosphosite-specific Signature Analysis,” *Molecular & Cellular Proteomics*, vol. 18, no. 3, pp. 576–593, Mar. 2019, doi: 10.1074/mcp.TIR118.000943.
