# Supplementary material for: BRAFV600E patient derived colon cancer organoids identify biomarkers of response to EGFR and BRAF inhibition and replicate clinical data
Source: J Exp Clin Cancer Res. 2026 Mar 25;45:92. doi: 10.1186/s13046-026-03699-2 (PMC13063721; doi:10.1186/s13046-026-03699-2)

Suppl. Figure 1

a

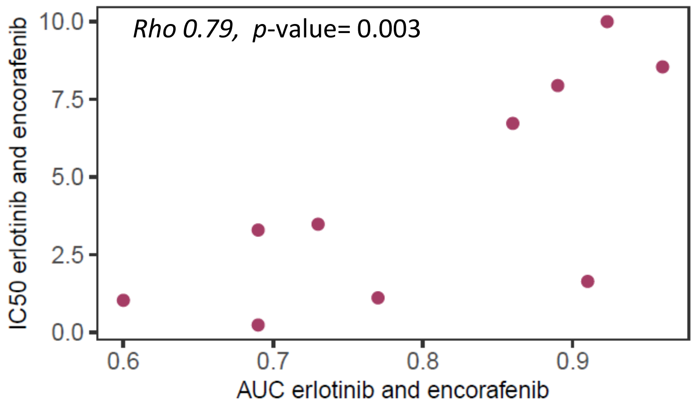

b

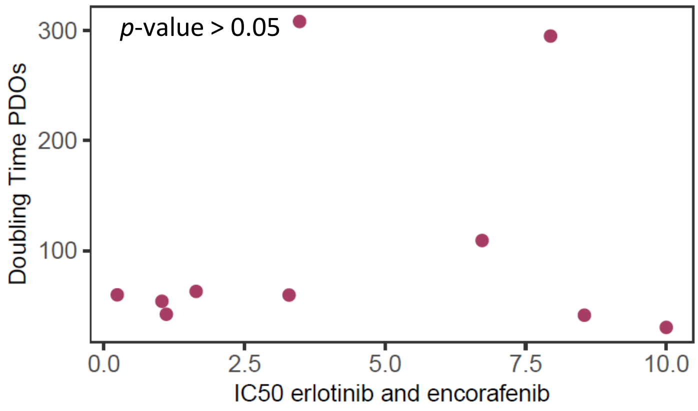

c

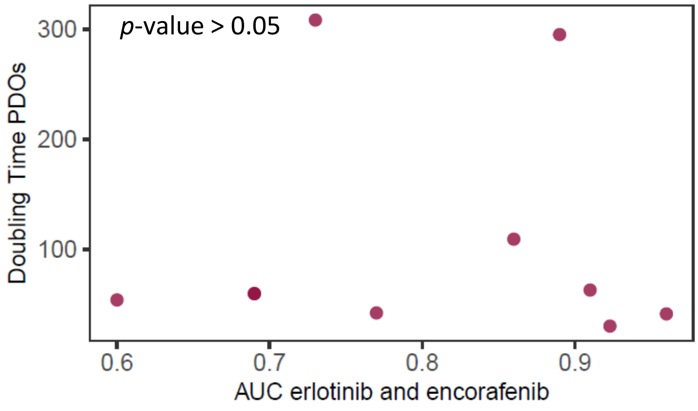

Suppl. Figure 2

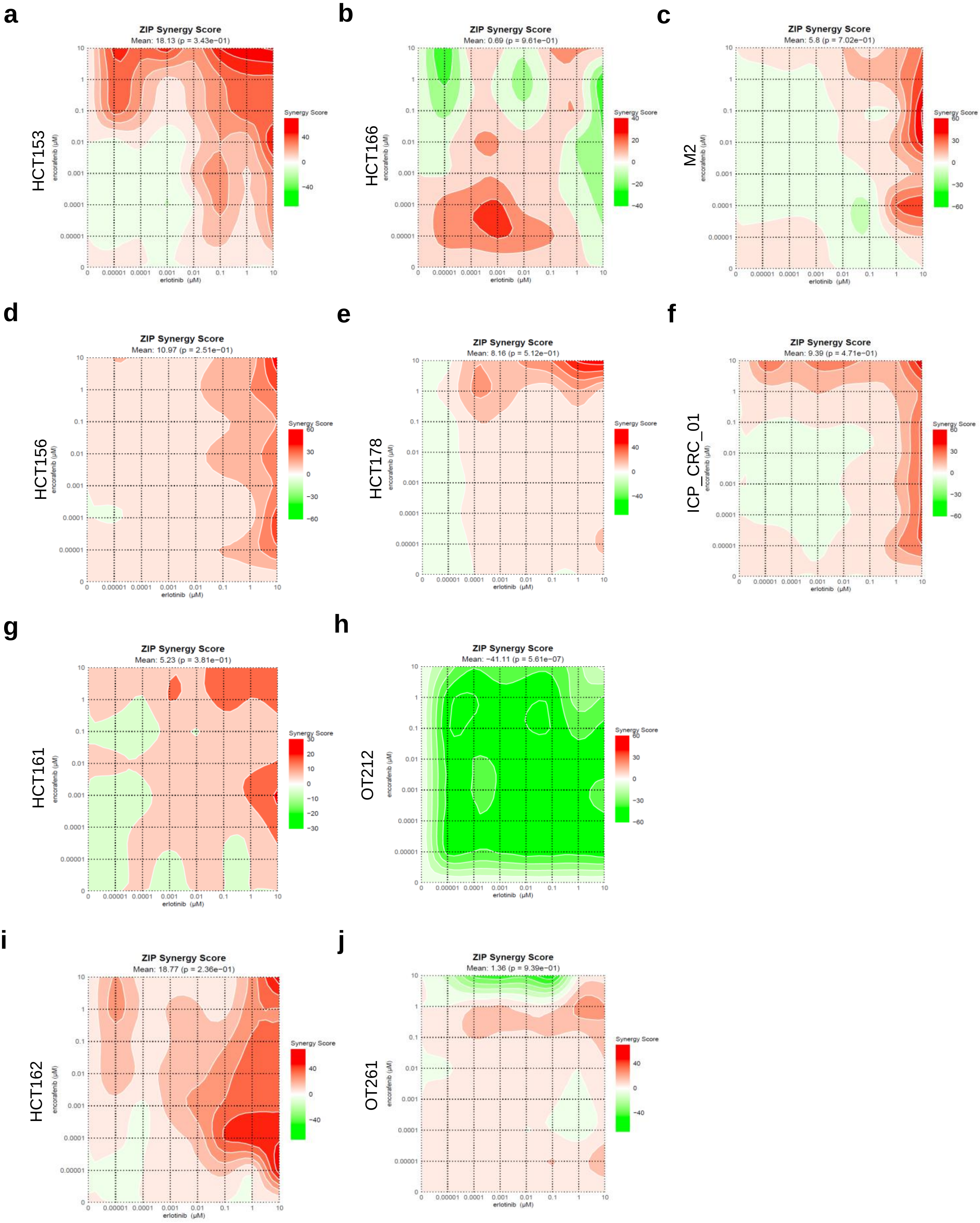

Suppl. Figure 3

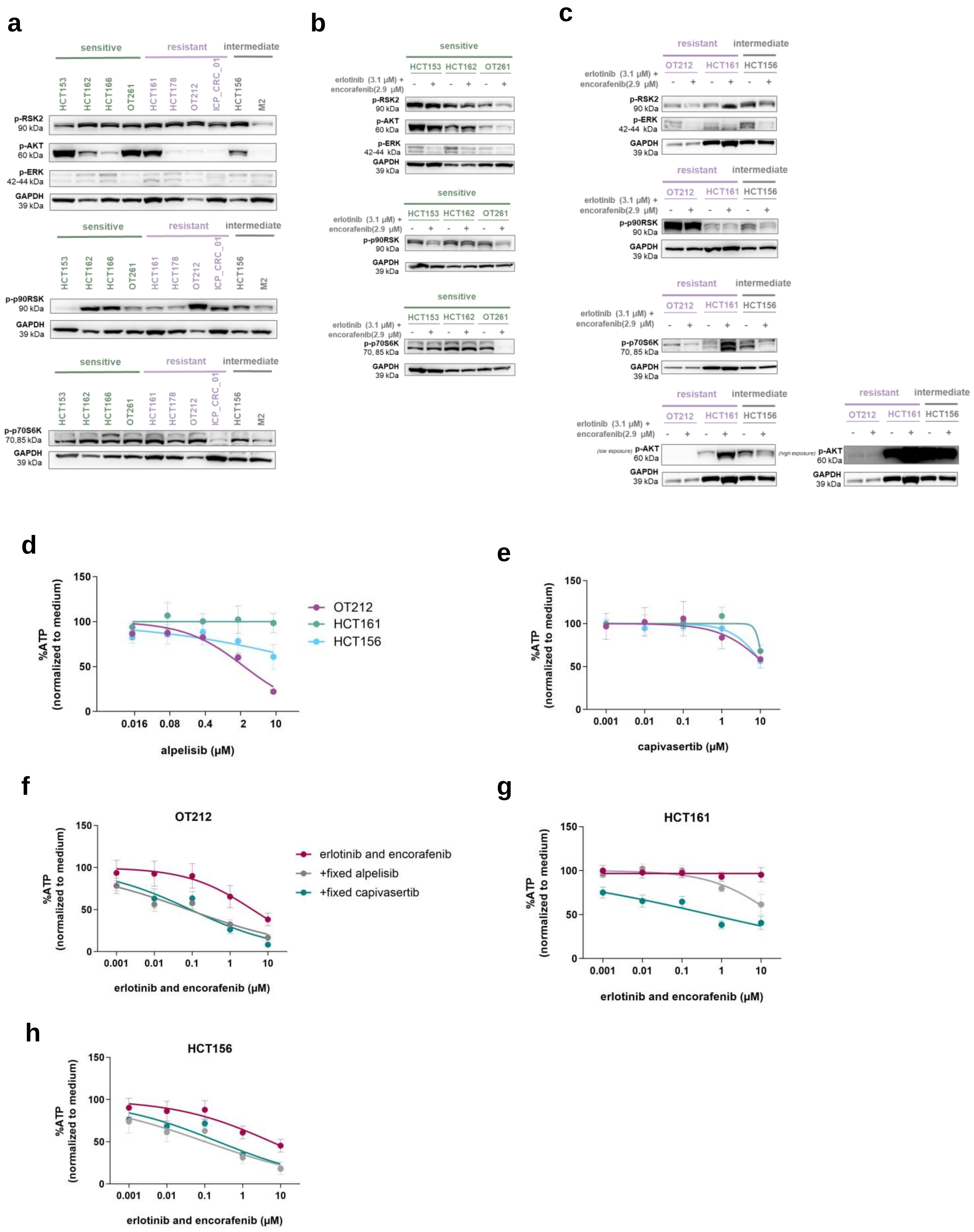

Suppl. Figure 4

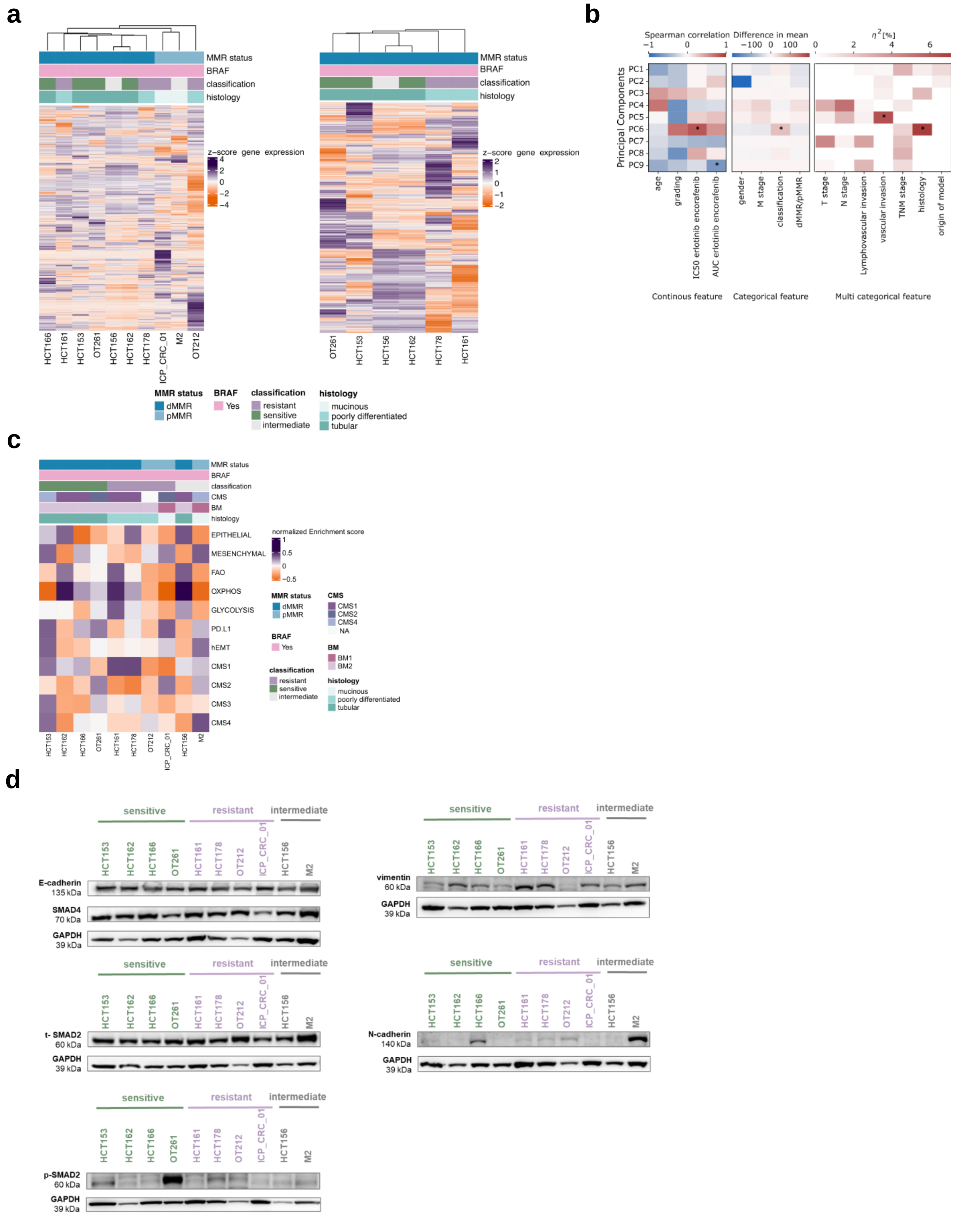

Suppl. Figure 5

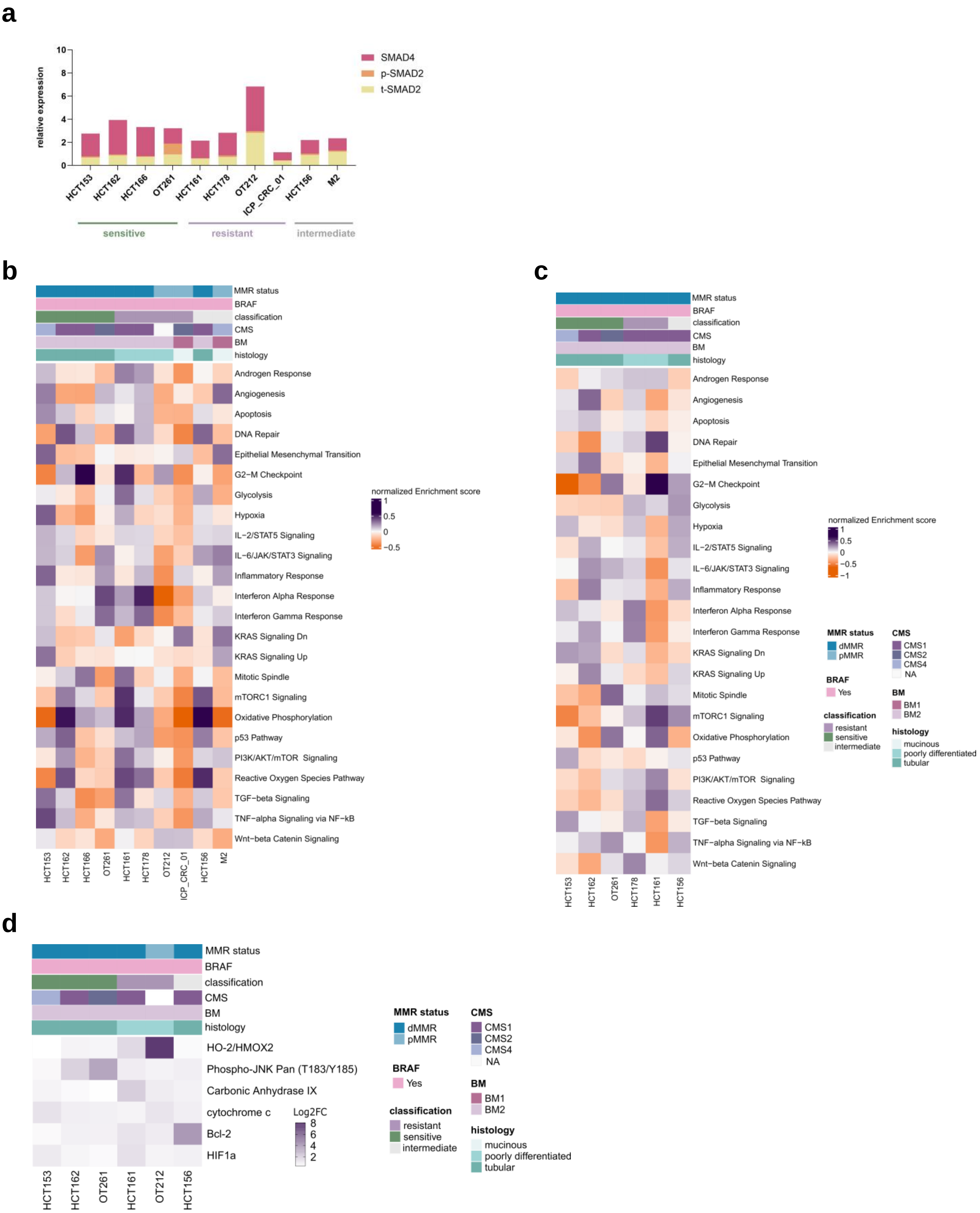

Suppl. Figure 6

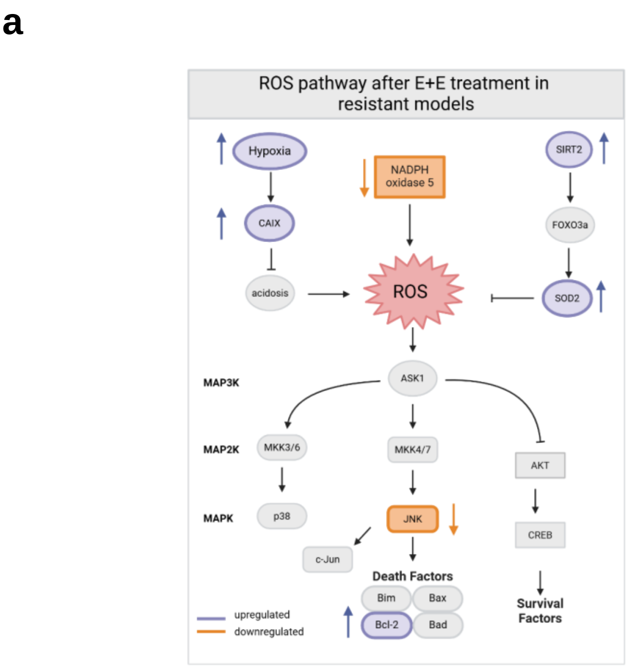

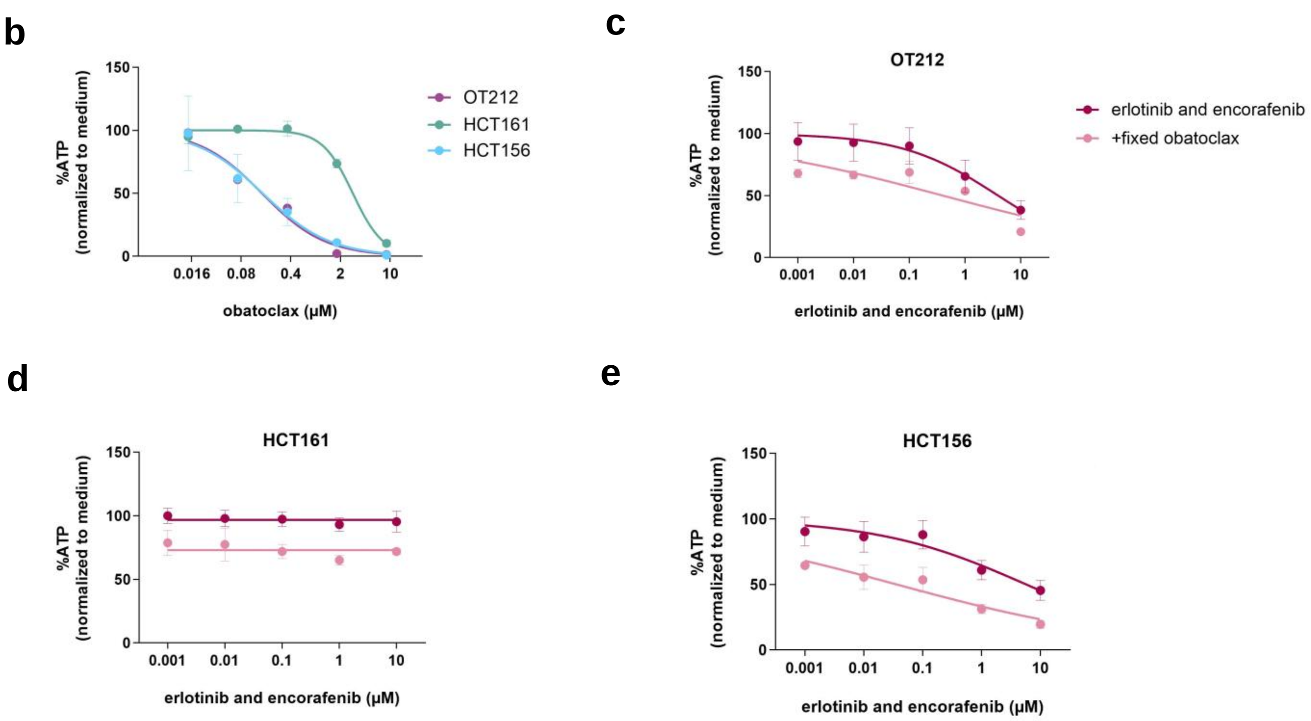

Suppl. Figure 7

a

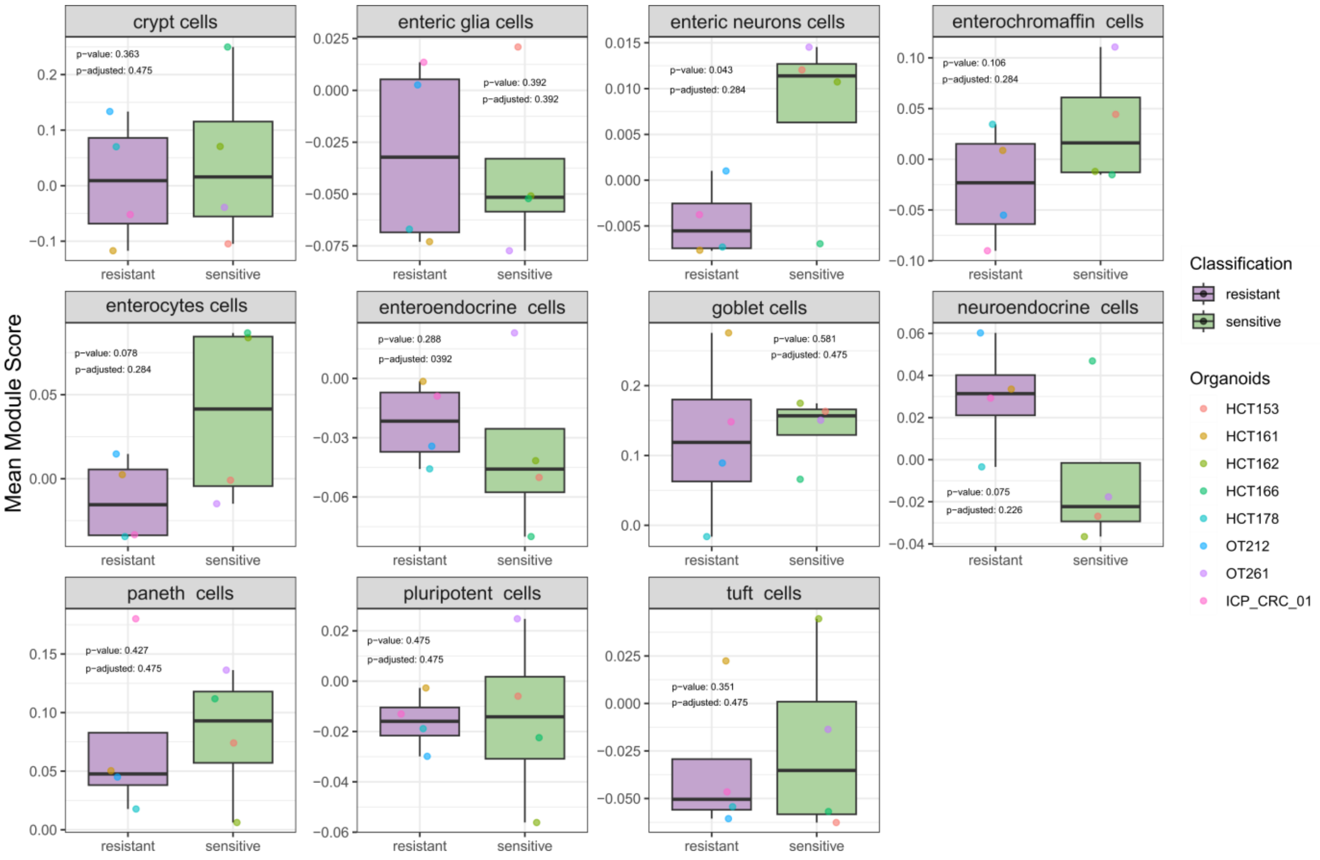

Supplement: Supplementary file 2 — Supplementary Material 2: Suppl. Figure 1. Correlations of Doubling Time, IC50 and AUC. a, Scatter plot showing strong correlation between IC50 and the AUC values of ten BRAFV600E PDOs treated with increasing doses of erlotinib and encorafenib (r = 0.74, p < 0.05, Pearson). b, Scatterplot showing no significant correlation between IC50 values of ten BRAFV600E PDOs treated with increasing doses of erlotinib and encorafenib and their doubling time (r = -0.12, p > 0.05, Spearman).c, Scatterplot showing no significant correlation between AUC values of ten BRAFV600E PDOs treated with increasing doses of erlotinib and encorafenib and their doubling time (r = -0.24, p> 0.05, Spearman). The models HCT166 and HCT156 have identical AUC and similar doubling time, thus letting their points to overlap and not being clearly evident in our graph. This is the reason why the scatter plot contains only nice points even if all ten models were included.Suppl. Figure 2. Synergy Scores of erlotinib and encorafenib in PDOs. (a-f) ZIP synergy scores of ten BRAFV600E PDOs treated with increasing doses of erlotinib or encorafenib. Values above 10 correspond to combinations that result in synergistic effects (red), values below -10 correspond to combination that result in an antagonistic effect (green) and values between 10 and -10 correspond to additivity. Suppl. Figure 3. Validation of mass spectrometry data. a, Western Blot of p-RSK2, p-P90, p-P70, p-AKT and p-ERK expression in ten BRAFV600E CRC PDOs at baseline. Models are annotated as sensitive (green), resistant (purple) and intermediate (grey). GAPDH was used as loading control. Blots are representative of at the least two biological replicates. b, Western Blot of p-RSK2, p-P90, p-P70, p-AKT and p-ERK expression in three sensitive models upon E+E treatment (3.1 µM erlotinib + 2.9 µM encorafenib). GAPDH was used as loading control. Blots are representative of at the least two biological replicates. c, Western Blot of p-RSK2, p-P90, p-P7 [file 13046_2026_3699_MOESM2_ESM.pdf]
